# Comparison and evaluation of statistical error models for scRNA-seq

**DOI:** 10.1186/s13059-021-02584-9

**Published:** 2022-01-18

**Authors:** Saket Choudhary, Rahul Satija

**Affiliations:** 1 New York Genome Center, 101 Avenue of the Americas, New York, 100013 USA; 2 Center for Genomics and Systems Biology, New York University, 12 Waverly Pl, New York, 10003 USA

**Keywords:** Single-cell RNA-seq, Normalization, Dimension reduction, Variable genes, Differential expression, Feature selection

## Abstract

**Background:**

Heterogeneity in single-cell RNA-seq (scRNA-seq) data is driven by multiple sources, including biological variation in cellular state as well as technical variation introduced during experimental processing. Deconvolving these effects is a key challenge for preprocessing workflows. Recent work has demonstrated the importance and utility of count models for scRNA-seq analysis, but there is a lack of consensus on which statistical distributions and parameter settings are appropriate.

**Results:**

Here, we analyze 59 scRNA-seq datasets that span a wide range of technologies, systems, and sequencing depths in order to evaluate the performance of different error models. We find that while a Poisson error model appears appropriate for sparse datasets, we observe clear evidence of overdispersion for genes with sufficient sequencing depth in all biological systems, necessitating the use of a negative binomial model. Moreover, we find that the degree of overdispersion varies widely across datasets, systems, and gene abundances, and argues for a data-driven approach for parameter estimation.

**Conclusions:**

Based on these analyses, we provide a set of recommendations for modeling variation in scRNA-seq data, particularly when using generalized linear models or likelihood-based approaches for preprocessing and downstream analysis.

**Supplementary Information:**

The online version contains supplementary material available at (10.1186/s13059-021-02584-9).

## Introduction

Single-cell RNA-sequencing (scRNA-seq) represents a powerful approach for the unsupervised characterization of molecular variation in heterogeneous biological systems [1, 2]. However, separating biological heterogeneity across cells that corresponds to differences in cell type and state from alternative sources of variation represents a key analytical challenge in the normalization and preprocessing of single-cell RNA-seq data [3, 4]. Upstream analytical workflows typically aim to achieve two separate but related tasks. First, data normalization aims to adjust for differences in cellular sequencing depth, which collectively arise from fluctuations in cellular RNA content, efficiency in lysis and reverse transcription, and stochastic sampling during next-generation sequencing [5]. Second, variance stabilization aims to address the confounding relationship between gene abundance and gene variance, and to ensure that both lowly and highly expressed genes can contribute to the downstream definition of cellular state. Although the use of unique molecular identifiers (UMIs), random sequences that label individual molecules, has been a promising approach to limit amplification bias [6, 7], variation due to sequencing depth still arises in such datasets and can be a major source of technical variance. These challenges are not unique to single-cell sequencing [8], but the sparsity of scRNA-seq data, coupled with substantial diversity in profiling technologies, necessitates the development and assessment of new methods.

While initial work focused on the development of cell “size-factors” for normalization, recent methods have been focused on the development and application of statistical models for scRNA-seq analysis. In particular, two recent studies proposed to use generalized linear models (GLMs), where cellular sequencing depth was included as a covariate, as part of scRNA-seq preprocessing workflows. Our sctransform [9] approach utilizes the Pearson residuals from negative binomial regression as input to standard dimensional reduction techniques, while GLM-PCA [10] focuses on a generalized version of principal component analysis (PCA) for data with Poisson-distributed errors. More broadly, multiple techniques aim to learn a latent state that captures biologically relevant cellular heterogeneity using either matrix factorization or neural networks [11–13], alongside a defined error model that describes the variation that is not captured by the latent space.

Together, these studies demonstrate the importance and potential of statistical models to assist in the normalization, variance stabilization, and downstream analysis of scRNA-seq data. However, such likelihood-based approaches require an explicit definition of a statistical error model for scRNA-seq, and there is little consensus on how to define or parameterize this model. While multiple groups have utilized a Poisson error model [10, 14–18], others argue that the data exhibit evidence of overdispersion, requiring the use of a negative-binomial (NB) distribution [5, 19–21]. Even for methods that assume a NB distribution, different groups propose different methods to parameterize their model. For example, a recent study [22] argued that fixing the NB inverse overdispersion parameter *θ* to a single value is an appropriate estimate of technical overdispersion for all genes in all scRNA-seq datasets, while others [23] propose learning unique parameter values for each gene in each dataset. This lack of consensus is further exemplified by the scvi-tools [11, 24] suite, which supports nine different methods for parameterizing error models. The purpose of this error model is to describe and quantify heterogeneity that is not captured by biologically relevant differences in cell state, and highlights a specific question: How can we model the observed variation in gene expression for an scRNA-seq experiment conducted on a biologically ‘homogeneous’ population?

## Results

### Shallow sequencing masks overdispersion in scRNA-seq data

We first explored whether a Poisson distribution was capable of fully encapsulating heterogeneity in scRNA-seq data that was independent of biological variation in the cellular state (i.e., “independent of the latent space” [25]). The rationale behind a Poisson model assumes that homogeneous cells express mRNA molecules for a given gene at a fixed underlying rate, and the variation in scRNA-seq results specifically from a stochastic sampling of mRNA molecules, for example due to inefficiencies in reverse transcription and PCR, combined with incomplete molecular sampling during DNA sequencing [5, 25]. The Poisson distribution constrains the variance of a random variable to be equal to its mean, and has been utilized for modeling UMI counts in multiple previous studies [15, 16]. While the Poisson distribution is well suited to capture variation driven by stochastic technical loss and sampling noise, it cannot capture other sources of biological heterogeneity between cells that are not driven by changes in cell state, for example, intrinsic variation caused by stochastic transcriptional bursts [26–28]. These fluctuations would cause scRNA-seq data to deviate from Poisson statistics, exhibiting overdispersion.

**Fig. 1:**
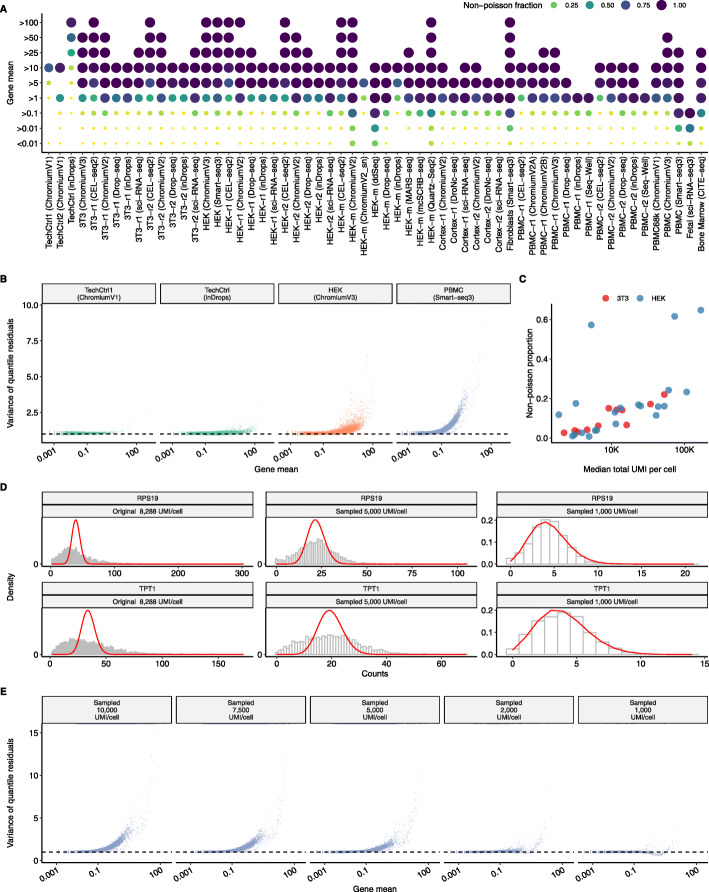
Shallow sequencing masks overdispersion in scRNA-seq data. **A** Proportion of genes that fail a goodness-of-fit test for a Poisson GLM (see the “Methods” section), as a function of gene abundance, for 59 scRNA-seq datasets. For visual clarity, both the color and diameter of each dot correspond to the fraction of genes that exhibit overdispersion. *Y*-axis represents non-cumulative gene abundance bins between two consecutive labels (for example, >1 refers to all genes with average abundance >1 UMI and ≤5 UMI). Values are listed in Additional file 1: Table S2. **B** Relationship between average gene abundance and quantile residual variance, after applying a Poisson GLM (see the “Methods” section). Results are shown for datasets profiling endogenous RNA (’‘technical controls”), a HEK293 cell line (’‘biological controls”), and human PBMC (’heterogeneous’). **C** In datasets profiling cell lines, the fraction of genes that exhibit overdispersion is correlated with average sequencing depth. **D** Distribution of molecular counts for highly expressed genes in the PBMC Smart-seq3 dataset after downsampling to two different sequencing depths. The expected density assuming a Poisson distribution is shown in red. **E** Same as (**B**) but after downsampling the PBMC Smart-seq3 dataset to five different sequencing depths

We therefore asked whether scRNA-seq data exhibited evidence of overdispersion by exploring the mean-variance relationship using technical controls (endogenous RNA and spike-ins), cell line (HEK293 and NIH3T3), and heterogeneous (PBMC, mouse cortex, fibroblasts) datasets profiled using multiple technologies (Additional file 1: Table S1). These datasets have varying sequencing depths with median UMIs per cell spanning from approximately 375 to more than 195,000 (Additional file 1: Figure S1). In each dataset, we performed a goodness-of-fit test, independently modeling the observed counts for each gene to be Poisson distributed, while accounting for differences in sequencing depth between individual cells (see the “Methods” section). For the technical control datasets [8, 14], where the input to each “cell” represented a uniform source of RNA, observed variation was largely consistent with the Poisson model (Fig. 1B). In contrast, when analyzing a human PBMC dataset profiled using Smart-seq3 [29], thousands of genes were poorly fit by a Poisson distribution (Fig. 1A and B), even after accounting for cell-to-cell variation in sequencing depth (Additional file 1: Table S2). While we expected to observe overdispersion for a subset of genes, particularly for those whose expression varies across multiple cell types, we were surprised to see that 97.6*%* of genes with average expression >1 UMI/cell failed the Poisson goodness-of-fit test. We observed a similar phenomenon when analyzing data from homogeneous HEK293 cells profiled with the 10X Chromium v2 system (HEK-r2; Fig. 1A and B), with 93% of genes exhibiting average abundance of >1 UMI/cell demonstrating evidence of overdispersion. In each of the 59 datasets we analyzed, genes exhibiting Poisson variation were overwhelmingly lowly expressed compared to genes that were overdispersed (Additional file 1: Figure S2). Moreover, when comparing results for cell-line datasets where we expect low levels of variation in cell state, we found that the global fraction of genes deviating from a Poisson distribution was correlated with the average sequencing depth of the dataset (Fig. 1C).

Our results suggest that scRNA-seq datasets commonly exhibit biological variation that exceeds Poisson sampling, but that the statistical power to detect these fluctuations requires sufficient sequencing depth. For example, when observing molecular counts in the deeply sequenced PBMC dataset (median 8288 UMI/cell), highly expressed genes such as TPT1 and RPS19 exhibited particularly strong deviations from Poisson variability (Fig. 1D). However, we found that when artificially downsampling the same dataset to 1000 UMI/cell, a depth that is common to shallowly sequenced scRNA-seq datasets, deviations from a Poisson distribution were strongly reduced (Fig. 1E). After downsampling, only 0.5*%* genes failed the Poisson goodness-of-fit test, demonstrating that reducing cellular sequencing depth can artificially create the appearance of Poisson variation. We conclude that the Poisson error model may represent an acceptable approximation for scRNA-seq datasets with shallow sequencing, but as the sensitivity of molecular profiling continues to increase, error models that allow for overdispersion are required for scRNA-seq analysis. Furthermore, we reiterate that the use of a Poisson error model does not account for the possibility of intrinsic stochastic noise in single-cell datasets, though this type of noise has been extensively described and does not correlate with changes in cell type or state.

### The level of overdispersion varies substantially across datasets

**Fig. 2:**
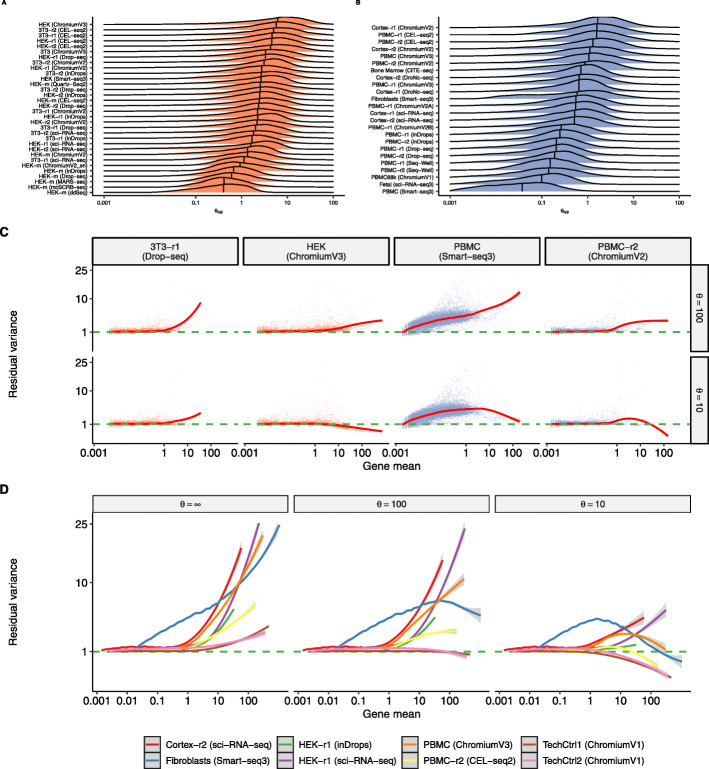
Overdispersion varies across datasets. **A**, **B** Distribution of per-gene values for the estimated inverse overdispersion *θ*_*NB*_ of a NB GLM across a range of cell lines (**A**) and heterogeneous datasets (**B**). We estimated parameters only for genes where the variance of counts exceeds the mean. Vertical bar indicates the median of the distribution, which varies substantially across datasets, but is concordant across replicate experiments within the same study. **C** Relationship between gene mean and the variance of Pearson residuals resulting from an NB GLM with *θ*=10 or *θ*=100. Each dot is a gene and the trendline (LOESS) is shown in red. **D** Same as (**C**), but shown for additional datasets and for *θ*=*∞* (Poisson). Only trendlines are shown for visual clarity

We next focused on the application of negative binomial error models, and considered different strategies for parameterizing the level of overdispersion associated with each gene. Recent work [22] suggested that a negative binomial model with a fixed parameterization (for example, inverse overdispersion parameter *θ*=100) could be applied to all scRNA-seq datasets to achieve effective variance stabilization. To explore whether a single value of *θ* could be applied to diverse scRNA-seq datasets, we first independently fit *θ* estimates for each gene in each dataset using a GLM with negative binomial errors (NB GLM), using library size as an offset to account for variation in cellular sequencing depth. We observed substantial differences in the magnitude of the estimated *θ* across different datasets, though replicate datasets from the same study yielded concordant results (Fig. 2A, B). Consistent with our previous results (Fig. 1B), *θ* values for each dataset varied across different biological systems, technologies, and sequencing depths.

In order to model overdispersion in scRNA-seq data, we focused on the use and parameterization of the negative binomial (NB) distribution. We note that while it is possible that overdispersion can also be accounted for using mixtures models or heavy-tailed distributions [30–32], the negative binomial distribution has been widely applied for the analysis of bulk RNA-seq data, and suggested for scRNA-seq analysis as well [19, 33–38]. To consider different methods for NB parameterization, we first tested the ability for a single value of *θ* to perform effective variance stabilization across a range of datasets. We processed each of our 59 datasets using an NB GLM after fixing *θ* to a single value for all genes in the dataset (for example, *θ*=100). We found that no single value of *θ* could achieve effective variance stabilization across all datasets. For example, a negative binomial error model with *θ*=100 resulted in clear heteroskedasticity in multiple datasets (Fig. 2C), as we observed a strong relationship between the mean expression of a gene, and its residual variance. This will artificially boost the weight of all highly expressed genes in downstream analysis such as dimensional reduction and clustering. We repeated the analysis with two alternative models, setting *θ*=*∞* and *θ*=10, both of which revealed similar shortcomings in multiple datasets (Fig. 2D and Additional file 1: Figures S3–S10). We conclude that fixing a single value of *θ* may achieve effective performance in certain cases, but is unlikely to generalize across the diversity of systems and technologies represented by scRNA-seq data.

### Gene overdispersion varies as a function of abundance

An alternative strategy for parameterizing *θ* leverages a well-characterized strategy for modeling counts in bulk RNA-seq data, where per-gene dispersion estimates have repeatedly been found to vary as a function of expression abundance [33, 34, 36, 37, 39–41]. In sctransform [9], we aim to estimate a global relationship between gene abundance and *θ* by employing a regularization procedure where parameters are first fit for each gene individually, but information from genes with similar average abundances is subsequently pooled together in order to improve the robustness of parameter estimates. The underlying rationale for this choice is the non-decreasing relationship between gene abundance and *θ* that has been repeatedly observed in bulk RNA-seq studies [33, 34, 36, 37, 39–41]. When analyzing each of the technologies and biological systems explored in this manuscript, we identified the same global patterns relating gene abundance and overdispersion levels (Additional file 1: Figures S11 – S14).

We also considered the findings from [22], which proposed that *θ* values should not vary as a function of gene abundance, and suggested that the relationship between these two variables was driven entirely by biases in the parameter estimation procedure, especially when analyzing lowly expressed genes. We first confirmed that lowly expressed genes, particularly those with average abundance <0.1 UMI/cell, posed difficulties for parameter estimation. This is because the vast majority of count values for these genes are 0, creating inherent challenges in maximum likelihood estimation. When estimating parameters on simulated data drawn from a negative binomial with fixed *θ*, we confirmed a bias for these genes that resulted in decreased parameter estimates for *θ* (Additional file 1: Figure S15). However, using two complementary analyses, we found that this bias was not sufficient to explain the true relationships we observed in biological data. First, we observed a non-decreasing relationship between gene abundance (*μ*) and dispersion (*θ*) even when moving beyond the threshold for lowly expressed genes, which we did not observe when analyzing simulated data (Additional file 1: Figure S16). Additionally, we attempted to increase (“upsample”) the depth of single cell datasets by pooling together molecular counts from cells with similar molecular profiles (see the “Methods” section) as inspired by the MetaCell framework [42]. We repeated the parameter estimation procedure on metacells generated either from single-cell data, or using our simulation framework (see the “Methods” section). Increasing the depth of sampling removed the effects of bias when analyzing simulated data, but preserved the observed relationship between *μ* and *θ* on real biological datasets (Additional file 1: Figure S16). We conclude that when modeling scRNA-seq data using a negative binomial distribution, the inverse overdispersion parameter *θ* does vary as a function of gene abundance, but the true nature of this relationship can be masked for genes with low molecular counts.

### Recommendations for modeling heterogeneity in scRNA-seq datasets

Our findings highlight how the extensive diversity of scRNA-seq datasets poses challenges in identifying uniform procedures for the preprocessing and normalization of scRNA-seq data. Sparsely sequenced datasets may appear to be compatible with the use of Poisson error models, but datasets with additional sequencing depth reveal clear evidence of overdispersion. The level of overdispersion, exemplified by the NB parameter *θ*, also can vary substantially across datasets, technologies, and systems and even varies within a dataset as a function of gene abundance. However, the estimation of robust parameter estimates for *θ* can be challenging for lowly expressed genes, especially when analyzing datasets with sparse sequencing. We therefore considered recommendations for balancing these considerations, providing the flexibility to learn error models that can be robustly applied to our full spectrum of scRNA-seq datasets.

We first recommend the use of negative binomial observation model as an alternative to the Poisson distribution. Our analyses show that the Poisson distribution is a reasonable approximation for technical-control datasets consisting of endogenous or spike-in RNA, as well as for some scRNA-seq experiments with shallow sequencing. However, scRNA-seq datasets from cell lines exhibit clear evidence of overdispersion at higher sequencing depths, even for genes that do not correlate with changes in cell type or state. At least some of this overdispersion likely originates from “intrinsic” noise, stochastic cellular variation that is inherent to the processes of mRNA transcription and degradation, and will affect the expression heterogeneity of all genes. While this variation is not a result of measurement error, it is not the primary focus of downstream scRNA-seq analyses, including the identification of cell types and states, and the inference of developmental trajectories. We therefore recommend that this variation be modeled independently of the latent space, which requires the use of a negative binomial error model. We note that the Poisson distribution is a special case of the negative binomial, and therefore the NB model can be successfully applied for datasets with very shallow sequencing, with appropriate parameter settings.

Second, we recommend learning negative binomial parameters separately for each dataset, rather than fixing them to a single value across all analyses. Moreover, we recommend allowing *θ* to vary across genes within a dataset, as a function of average gene abundance. The relationship between *μ* and *θ* has been repeatedly demonstrated in bulk RNA-seq and is apparent across diverse scRNA-seq datasets as well, particularly for genes with sufficient sequencing depth. Using a fixed *θ* to parameterize all genes in a scRNA-seq dataset leads to ineffective variance stabilization and results in a global relationship between expression level and expression variance (Fig. 2 and Additional file 1: Figures S3 and S4). We note that the recommendations described above relate not only to GLM-based preprocessing workflows, but also probabilistic or likelihood-based models [11, 24, 43].

**Fig. 3:**
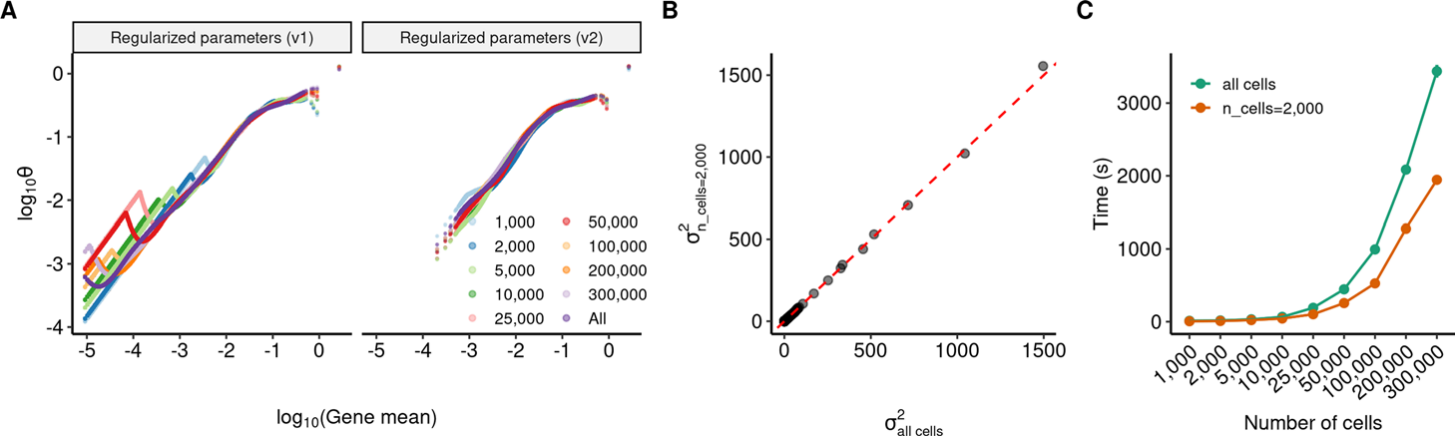
A modified regularization procedure improves the robustness of parameter estimates. **A** Left: Estimated parameter estimates for *θ* on the Fetal sci-RNA-seq3 dataset [76], using the original regularization procedure from [9] (v1 regularization). Regularized estimates were learned using all cells (purple line), or downsampled cell subsets. Right: Same as (**A**), but using a modified procedure where the GLM slope was fixed, and genes where *σ*^2^≤*μ* and *μ*<0.001 were excluded from regularization (v2 regularization) which improves robustness, and enables us to learn parameter estimates from a subsample of 2,000 cells. **B** Correlation of Pearson residual variance after applying a NB GLM with v2 regularization where parameters were estimated from all 377,456 cells (*x*-axis), and a subsample of 2000 cells (*y*-axis). **C** Green curve: total sctransform run time as a function of dataset size, using all cells to estimate parameters. Orange curve: total runtime when using a subsample of 2000 cells, which increases computational efficiency for large datasets

Our analyses highlighted that lowly expressed genes with particularly sparse molecular counts often lacked sufficient information content to robustly detect overdispersion. We therefore designed a modified regularization procedure for learning GLM parameter estimates and calculating Pearson residuals (see the “Methods” section). First, following the recommendations from [22], we fix the slope of the NB GLM to its analytically derived solution of ln(10), so that only the overdispersion and intercept parameters are estimated for each gene. Second, we reasoned that for genes with very low expression (*μ*<0.001), or where the variance of their molecular counts does not exceed the mean (i.e., *σ*^2^≤*μ*), we do not have sufficient evidence for overdispersion to fit negative binomial parameters. We therefore removed these genes from the regularization process and fixed their *θ* parameter to *∞*, exemplifying a Poisson distribution. For example, in the scRNA-seq dataset of HEK cells profiled with SMART-Seq3, we removed 1577 genes (8.5*%*) at this stage, the majority of which were lowly expressed (66.64*%*<0.1 UMI/cell). We found that our modified regularization enables us to reproducibly learn gene-specific parameters even when using a subset of cells in the estimation procedure. This indicates increased robustness (Fig. 3A) and allows us to learn a regularized relationship between *μ* and *θ* using only a subset of cells that achieves nearly identical results (Fig. 3B) with increased computational efficiency (Fig. 3C). Third, we apply a lower bound on the minimum variance while calculating the Pearson residual for each per cell to prevent genes with minimal information content from resulting in high residual variance (see the “Methods” section). In particular, this step helps to ensure that very low UMI counts (i.e., 1 to 2 molecules) are not assigned extremely high Pearson residuals (Additional file 1: Figure S17). We have implemented our findings and the updated regularization procedure as a version 2 update of sctransform (sctransform v2).

**Fig. 4:**
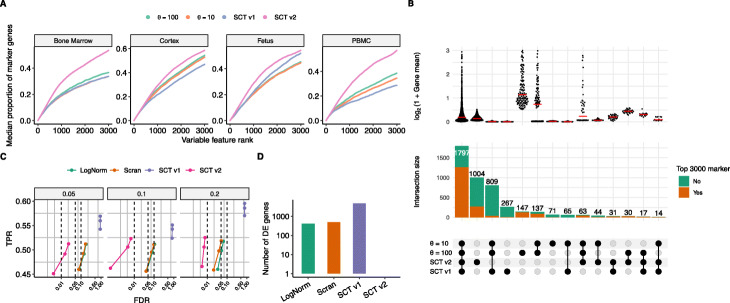
Benchmarking on variable feature selection and differential expression analysis. **A** Proportion overlap (median) of cluster marker genes and identified variable features using fixed *θ*={100,10}, sctransform v1, and sctransform v2. Marker genes were identified using presto [65], based on unsupervised clustering of log normalized data. Additional plots are shown in Additional file 1: Figure S22. **B** Comparison of variable features selected by *θ*={100,10} and our v1 and v2 regularization procedure on a PBMC (ChromiumV3) dataset. The bottom sub-panel represents the top 3000 variable genes identified by four different methods, and groups genes into categories based on the methods where they were identified. Top sub-panel shows the distribution of logarithmic gene mean within each category, with the median value marked in red. Middle sub-panel shows the number of genes within each category, and their overlap with cluster markers. **C** Benchmarking differential expression analysis. Observed overall true-positive rate (TPR) and false discovery rate (FDR) values for DE genes at FDR cutoffs of 1%, 5%, and 10% using a Wilcoxon rank-sum test (Additional DE methods are indicated in Additional file 1: Figure S23). Dashed vertical lines indicate desired FDRs. Methods that control FDR at their desired level should fall to the left of the corresponding dashed line. Performances were averaged across three simulation replicates. Data was simulated with muscat [79] using three annotated cell types (CD4 T, monocytes, and natural killer cells) from a Smart-seq3 and a Drop-seq PBMC dataset. Titles indicate simulated proportion of DE genes. **D** Number of differentially expressed genes identified between two groups of biological identical NK cells (PBMC Smart-seq3) where one group was randomly downsampled to 20% sequencing depth. Additional DE methods are indicated in Additional file 1: Figure S24. SCT = sctransform; LogNorm = standard log-normalization

To test the broad applicability of this procedure, we applied it to each of the 59 datasets examined in this manuscript. In each case, we achieved effective variance stabilization as we observed no global relationship between gene expression levels and the variance of the resulting Pearson residuals (Additional file 1: Figures S18 - S21). We also quantitatively benchmarked the ability of our procedure to select highly variable genes, which is an essential step in downstream analysis such as dimensional reduction and clustering. To evaluate the procedure’s effectiveness, we employed a metric inspired by [44] and calculated the overlap of highly variable genes with the list of marker genes identified using unsupervised clustering analysis (see the “Methods” section). Across all systems, highly variable genes identified using sctransform v2 had higher overlap with the marker genes (Fig. 4A and Additional file 1: Figure S22). For example, in PBMC datasets, a median of 1712 of the first 3000 variable genes detected by sctransform v2 are also among the top 3000 marker genes (Additional file 1: Table S3). On the other hand, variable genes detected by fixing *θ*=10 or *θ*=100 result in 1155 and 1035 genes, respectively, in the top 3000 markers list while sctransform v1 captures only 860 marker genes. Importantly, genes selected by sctransform v2 spanned a wide range of expression levels. In contrast, sctransform v1 exhibited a bias towards non-informative genes with extremely low expression levels, while negative binomial models with fixed *θ*={10,100} demonstrated biased selection towards genes with very high average abundance (Fig. 4B).

As an additional benchmark, we compared the ability of sctransform v2 to leverage its corrected UMI counts after normalization (see the “Methods” section) to identify differentially expressed (DE) genes. We used the simulation and benchmarking framework introduced by muscat [45], which found that the performance of differential expression tasks was dependent on upstream normalization. In the original sctransform manuscript, we proposed performing differential expression analyses directly on the Pearson residuals. While this approach successfully identifies DE genes within a single dataset, even when cell populations vary in their sequencing depth, it generates a high proportion of false positives when performing differential expression across datasets that have been normalized independently [45]. We reasoned that performing differential expression on corrected UMI counts across datasets (see the “Methods” section) could alleviate this problem.

We used muscat to generate a simulated scRNA-seq where cells originated from two different conditions, with a varying of genes that were known to be differentially expressed (DE) across these conditions. We then performed normalization using both GLM-based (sctransform v2, sctransform v1) and size-factor based (scran [46], standard log-normalization), calculated differentially expressed genes across conditions, and compared the results to ground-truth. We found that sctransform v2 resulted in higher true positive rate (TPR) for the same false discovery rate (FDR) threshold for varying proportion of DE genes using the Wilcoxon [47] rank sum test (Fig. 4C) or MAST [48] based test (Additional file 1: Figure S23). We also used DESeq2 [37], a method developed for identifying differentially expressed genes in bulk RNA-seq data, to identify DE genes in the simulated scRNA-seq data. With raw counts as input, DESeq2 performed similarly to sctransform v2, with optimal performance in cases where 20*%* of genes were differentially expressed.

As a complementary analysis, we took a subset of cells (305 cells from a PBMC Smart-seq3 dataset) and artificially created a new dataset by downsampling each cell to 20% of its original UMI counts, and normalized both the original and downsampled datasets independently. These two groups of cells are biologically identical, but differential expression performed using sctransform v1 yielded 4,747 DE genes. Log-normalized and scran-normalized values resulted in 415 and 498 differentially expressed genes, respectively (adjusted p-value <0.05; Fig. 4D). Sctransform v2 detected no differential genes between the two subpopulations. Using DESeq2 with raw counts resulted in 9 differentially expressed genes, while when run with corrected counts, it resulted in no DE genes (Additional file 1: Figure S24). Additionally, we applied the same testing strategy but without simulated downsampling on a real dataset of HEK293 cells profiled using Quartz-Seq2 and Drop-seq where sctransform v2- based normalized counts resulted in the lowest false positives (<1600) independent of the choice of DE test (see the “Methods” section and Additional file 1: Figure S25). Contrastingly, DESeq2 with raw counts marked 12,270 genes as differentially expressed, while scale factor-based normalization methods resulted in >8500 DE genes. We conclude that sctransform v2- based normalization enables effective differential analysis across datasets and minimizes false-positive results even when there are significant differences in sequencing depth across experiments and conditions. Taken together, our results demonstrate that sctransform v2 not only best represents the statistical properties of scRNA-seq data, but it also improves performance on key downstream analyses including variable gene identification and differential expression.

## Discussion

The application of statistical count models for preprocessing of scRNA-seq data overcomes important challenges that cannot be addressed by using linear size or scaling factor-based normalization. However, these techniques require the selection of an appropriate error distribution and accompanying parameter settings. Here, we explore these questions through the analysis of a wide diversity of scRNA-seq datasets varying across technologies, biological systems, and sequencing depths. We have implemented our findings, along with an improved procedure for estimating model parameters and calculating Pearson residuals, in a version 2 update of sctransform. Sctransform v2 performs effective variance stabilization across a wide range of scRNA-seq datasets and improves downstream performance for variable gene identification and differential expression analysis.

Our analyses revealed three key insights. First, we found that all scRNA-seq datasets exhibited clear evidence of overdispersion (i.e., deviation from a Poisson distribution), even after accounting for differences in sequencing depth, once exceeding a minimum expression level. This threshold varied across datasets as a function of average sequencing depth. This result strongly supports the use of negative binomial error models when analyzing UMI datasets. Second, we found that the negative binomial overdispersion parameter *θ* varied substantially across datasets, arguing against the use of a fixed *θ* estimate. Finally, we found that all datasets exhibited a dependence between gene abundance and overdispersion estimates. This result is robust even when considering potential biases in the overdispersion parameter estimation, and supports an empirical approach to learn regularized parameter estimates, as is commonly performed in bulk RNA-seq analysis.

Taken together, these results are compatible with the idea that cell-to-cell variation in scRNA-seq count data can be decomposed into multiple broad categories. The first represents variation in cell type and state which is biologically driven and encoded in cellular transcriptomes. This heterogeneity can be observed as covariation in the expression pattern of multiple genes and is the primary interest and focus of downstream analysis, and is typically represented in a latent space that can be learned via linear or non-linear dimensional reduction techniques. A second source represents technical measurement error arising from the stochastic loss of molecules during library preparation and sequencing. This sampling error can be modeled using a Poisson distribution and, particularly for shallowly sequenced datasets, represents a substantial source of remaining heterogeneity.

In addition, fluctuations in gene expression are also driven by noise that is inherent to the processes of mRNA transcription and degradation (i.e., “intrinsic noise”) and manifests as overdispersion in scRNA-seq data. The presence of intrinsic noise has been extensively characterized and is an inevitable consequence of the gene regulatory process [26, 27, 49, 50]. This heterogeneity manifests as variations within the same gene that arise from stochastic biochemical events. These variations are known to be modulated in a gene-specific manner by genetic [51, 52] and epigenetic factors [53, 54] as well as translational events [55]. Therefore, no two cells can generate mRNA molecules at exactly the same rate (an assumption of a Poisson process), even if they originate from the same “homogeneous” population. Our analyses demonstrate that intrinsic noise is readily detectable for genes with sufficient sequencing depth, but can be masked in shallow datasets (Fig. 1E). While intrinsic noise is not driven by measurement error, it should also be modeled independently of the latent space. Therefore, as the sensitivity and depth of scRNA-seq datasets continue to increase, the use of negative binomial error models will become increasingly important. Moreover, the level of intrinsic noise can vary across biological systems and gene abundance levels, motivating the use of a data-driven regularization procedure to learn gene-level overdispersion parameters.

## Conclusions

Our analyses highlight the importance of considering a diversity of datasets when evaluating the statistical properties of new data types. While our results are therefore applicable to scRNA-seq measurements, they cannot be directly applied to new single-cell modalities, including protein measurements (i.e., CITE-seq [56]), chromatin accessibility profiles (i.e., scATAC-seq [57]), and DNA interaction maps (i.e., scCUT&TAG [58, 59]). As with cellular transcriptomes, these modalities can be profiled using multiple technologies that vary in their sensitivity and sparsity. We anticipate exciting future work that will characterize and parameterize heterogeneity in these data types, to achieve effective preprocessing and normalization.

## Methods

### Data sources and preprocessing

All datasets were obtained as preprocessed count matrices from Gene expression omnibus (GEO), EBI ArrayExpress, or author’s website. In each case, we utilized cells that had passed the QC thresholds set by the original study authors. However, to minimize the effect of potential cell outliers in our data, we filtered out cells that fell outside of the 5 to 95% UMI quantiles in each dataset. Additionally, we removed all cells where more than 15% of reads mapped to mitochondrial transcripts. We did not perform any filtering for the Fetal sci-RNA-seq3 dataset as it had already been filtered and annotated by the authors. The dataset source and associated publication are available in Additional file 1: Table S1.

### Goodness of Fit test using a Poisson GLM

To explore whether a Poisson distribution represents an appropriate error model for UMI-based scRNA-seq data, we fit a Poisson GLM adjusting for differences in library size modeled as an offset. To reduce the computational complexity, we subsampled 1000 cells in a density dependent manner from the count matrices of each dataset: the probabilty of selecting a cell *c* is $\frac {1}{d(\log _{10} N_{c})}$, where *d* is the density estimate of all log10-transformed total cell UMIs and *N*_*c*_ is the total UMI counts in cell *c*. These subsampled count matrices were then used to fit a Poisson GLM for each gene UMI vector with total UMI content of each cell modeled as an offset vector (glm.fit(gene_umi ∼1, offset=log(total_umi), family=Poisson(link=~log~)) in R. We then performed a goodness of fit test on the randomized quantile residuals [60] of this GLM model fit calculated using statmod::qresid(model). If the data is well-described by the model, the sum of squares of the quantile residuals is expected to follow a chi-squared distribution with degrees of freedom = *N*_cells_−1 where *N*_cells_ represents the total number of cells in the dataset. We chose quantile residuals to measure the goodness of fit, as they have lower type-I error and higher power in comparison to other residuals for identifying misspecification [61]. To calculate *p*-values, we used the pchisq function in R (pchisq(qresid, df=model$df.residual, lower.tail=FALSE)). To control for multiple testing, we adjusted *p*-values using the qvalue method available through the qvalue package [62]. We used a *q*-value threshold of 0.01 to accept or reject the fit to the Poisson model. Library sizes reflected in Fig. 1E were calculated based on the subset count matrices.

### Assessing overdispersion after downsampling sequence depth

In Fig. 1D, E we assess the level of dispersion in the PBMC Smart-seq3 dataset, after downsampling the dataset to different sequencing depths. The full dataset contains 2629 cells with a median UMI/cell of 8288 with a maximum coverage of 20,463 UMI/cell. When downsampling to 10,000 UMI/cell, we first excluded 1837 cells where <9900 UMIs were detected in the dataset. For the remaining cells, we randomly sampled molecules at a proportion expected to yield 10,000 UMI/cell on average and retained only cells that contained UMIs in the range 10,000±100 to minimize the effect of differences in sequencing depth. We repeated this process for multiple sequencing depths shown in Fig. 1D, E.

### Comparing levels of overdispersion across datasets

In Fig. 2A, B, we fit NB GLM to each gene in each dataset, in order to estimate the inverse overdispersion parameter *θ*. We model the observed counts for each gene using the following model gene_umi ∼1, and estimate parameters using glmGamPoi::glm_gp(gene_umi, model, offset=log(total_umi), size_factors=FALSE) using the glmGamPoi package [63]. We perform this procedure for all genes where the variance of the observed counts exceeds the mean.

### Modeling scRNA-seq datasets with sctransform

For clarity, we restate the modeling framework used in sctransform [9]. In sctransform, UMI counts across cells in a dataset are modeled using a generalized linear model (GLM). The total UMI count per cell is used as an offset in the GLM. Thus, for a given gene *g* in cell *c*, we have

$$\begin{array}{*{20}l} x_{gc} &\sim \text{NB}(\mu_{gc}, \theta_{g})\\ \ln \mu_{gc} &= \beta_{g0} + \ln n_{c}, \end{array} $$

where *θ*_*g*_ is the gene-specific dispersion parameter, $n_{c}=\sum _{g} x_{gc}$ is the total sequencing depth and the variance of the negative binomial (NB) is given by $\mu _{gc} + \mu _{gc}^{2}/\theta _{g}$.

We perform three steps to remove technical noise and perform variance stabilization. In the first step, the inverse overdispersion parameter *θ* is individually estimated using a subset of genes (2000 by default), which are sampled in a density-dependent manner according to their average abundance. In the next step, we calculate a smoothed curve that characterizes the global relationship between *μ* and *θ*, thereby regularizing *θ* estimates as a function of gene mean. We perform the same regularization for the intercept parameter. We use the geometric mean to estimate gene abundance, which is more robust to outlier values in scRNA-seq. As outlier values can originate from multiple sources including the presence of cell doublets, errors in UMI collapsing, or ambient RNA, we have empirically improved performance when using the geometric mean instead of the arithmetic mean. Although sctransform supports multiple estimators for *θ*, we recommend the use of glmGamPoi [63], an alternate estimator that is more robust and faster.

In the final step, we use the regularized parameter estimates to calculate Pearson residuals *Z*_*gc*_. For each gene-cell combination, the Pearson residuals *Z*_*gc*_ are given by

$$\begin{array}{*{20}l} Z_{gc} &= \frac{x_{gc}-\mu_{gc}}{\sigma_{gc}}\\ \mu_{gc} &= \exp{\beta_{g0} + \ln{n_{c}}} \\ \sigma_{gc} &= \sqrt{\mu_{gc} + \frac{\mu_{gc}^{2}}{\theta_{gc}}}, \end{array} $$

The “residual variance” for a gene represents the remaining variation in gene expression that is not explained by the sctransform model, and is defined as:

$$\begin{array}{*{20}l} \text{residual variance}_{g} &= \frac{1}{C-1}\sum_{c=1}^{C} \left(Z_{gc} - \bar{Z_{g}}\right)^{2}\\ \bar{Z_{g}} &= \sum_{c=1}^{C} Z_{gc}, \end{array} $$

where *C* represents the number of total cells in the dataset.

### Evaluating the performance of a GLM with fixed *θ*

In Fig. 2C, D and Additional file 1: Figures S3–S10, we model each of the scRNA-seq datasets using a NB GLM with a fixed value of *θ* for each gene in each dataset. To test this, we utilize the “offset” model as described by Lause et al. in [22]. We repeated the analysis with three different values for the fixed overdispersion parameter, *θ*=*∞*,*θ*=100, and *θ*=10.

### Improving the robustness of parameter regularization

In Fig. 3, we explore a modified regularization procedure to improve the robustness of NB parameter estimates, particularly for lowly expressed genes, and to increase computational efficiency. We make two changes to the estimation procedure described in [9]. First, we fix the slope parameter of the GLM to ln(10) with log10(total UMI) used as the predictor. As described in [22], this value represents the analytically derived solution for this parameter and closely mirrors the regularized values we had obtained for the slope parameter in the original sctransform procedure. While [22] also recommends fixing the intercept parameter for the GLM, an approximate solution to the maximum likelihood estimate of this parameter can only be obtained for large values of *θ*. As our data-driven estimates for *θ* demonstrate that this parameter can vary substantially across datasets, we continue to set the intercept parameter for the GLM through regularization.

As a second modification, we remove a subset of genes prior to performing regularization. In particular, we reasoned that for genes with either very low abundance (*μ*<0.001), or where the variance of count values did not exceed the average abundance (i.e., *σ*^2^≤*μ*), we lacked sufficient information to learn robust NB parameter estimates. We therefore exclude these genes from the regularization procedure and set their *θ* parameter estimates to *∞* for all downstream analyses. We note that this filtration step occurs rapidly, as the per-gene mean and variance can be efficiently calculated. For this filtration step, we use the arithmetic mean to set abundance, as this value should be compared with gene variance to determine evidence of overdispersion. For these genes, the regularized intercept ($\hat {\beta }^{\text {poisson}}_{g0}$) is set to the analytically derived solution from [22], with a fixed slope of ln(10):

As a third modification, we placed a lower bound on gene-level standard deviation when calculating Pearson residuals. For some genes with extremely low expression, our previous approach would result in a high Pearson residual even with only 1-2 UMI detected, as the expected mean and standard deviation per cell are very small (Additional file 1: Figure S17A–D). When calculating Pearson residuals, we therefore set the minimum standard deviation to $\frac {\text {nzmedian}}{5}$ where nzmedian is the median calculated using only the non-zero observed UMIs. For most datasets, nzmedian represents 1 UMI, ensuring that cases where only a single UMI is detected do not result in a Pearson residual greater than 5. We found that this procedure helped to remove genes with extremely low abundance from being spuriously identified as highly variable (Fig. 4B and Additional file 1: Figure S17E–F).

$$\begin{array}{*{20}l} \hat{\beta}^{\text{poisson}}_{g0} & = \ln \left(\sum_{c}x_{gc}\right) - \ln \left(\sum_{c} n_{c} \right) \end{array} $$

### Simulation of UMI counts with fixed overdispersion

To explore the potential bias of maximum-likelihood (ML) estimators, we simulated an scRNA-seq dataset with fixed levels of overdispersion. We fixed *θ* to different values {0.001,0.01,0.1,1,10,100}, and simulated scRNA-seq counts from an NB distribution, using gene means that were taken from the PBMC Smart-Seq3 dataset. We next estimated parameter values for *θ* using both the v1 and v2 versions of our sctransform regularization procedure using glmGamPoi [63] as an estimation engine. We also estimated a maximum likelihood of *θ* using glmGamPoi without explicitly accounting for library size (MLE). To create an “upsampled” dataset where the sequencing depth is higher, we multiplied the estimated means *x*_*gc*_ by a factor of 500 and repeated the sampling procedure.

### Increasing sequencing depth by creating metacells

In order to “upsample” the PBMC Smart-seq3 dataset, we ran MetaCells v0.3.5 [42] for three different values of “K” parameter (200, and 300, and 400) with all other parameters as defaults. UMI counts of cells belonging to one metacell were consolidated to create a metacell count, resulting in a higher sequencing depth. These metacells were then used as input to sctransform to estimate per gene *θ*.

### Marker overlap analysis

In order to define a ground truth for comparing the effectiveness of variable gene selection procedure, we determined marker genes for each dataset by performing unsupervised clustering on log-normalized data using 2000 variable features selected using Seurat v3 [64] variable feature selection strategy. We then identified marker genes for each of the identified cluster using presto [65]. To shortlist the *top* 3000 marker genes, we removed genes with *p*-value >0.05 and average log-foldchange <0.25 and then selected the top 3000 genes with the highest log-foldchange. Variable genes selected by each method were compared against this list. The UpSet [66] plot in Fig. 4B was generated using the ComplexUpset package [67].

### Correcting counts by regressing out sequencing depth

While the primary output of the sctransform procedure is a set of Pearson residuals, we can also estimate “corrected” counts for each gene in a cell. These corrected counts should no longer exhibit technical variation driven by differences in sequencing depth and can be used for downstream visualization and differential expression analyses in sctransform v2. Corrected counts are obtained by setting the sequencing depth for all the cells to a fixed value and reversing the learned regularized negative-binomial regression model. For a given Pearson residual (*Z*_*gc*_) calculated using the regularized parameterization approach, the corrected counts (*y*_*gc*_) can be estimated for each gene per cell as if all the cells have been sequenced to the same sequencing depth *n*_0_:

$$\begin{array}{*{20}l} \mu_{gc} &= \exp{\beta_{g0} + \ln{n_{0}}}, \\ \sigma_{gc} &= \sqrt{\mu_{gc} + \frac{\mu_{gc}^{2}}{\theta_{gc}}},\\ y_{gc} &= \text{floor}(Z_{gc} \sigma_{gc} + \mu_{gc}), \end{array} $$

where the floor operation rounds *y*_*gc*_ to the nearest lower non-zero integer. By default, *n*_0_ is set to the median sequencing depth of the dataset.

### Differential expression analysis

We performed differential expression using DESeq2 [37], MAST [48], and Wilcoxon rank-sum test [47]. For DESeq2, we used raw or sctransform v2 corrected counts as input and for estimating the size factors we used scran::computeSumFactors with useT=TRUE, minmu=1e-6, and fitType=~glmGamPoi~ following the recommendations in DESeq2 vignette [68]. MAST and Wilcoxon rank-sum test were run using pearson residuals (sctransform v1), log-normalized, scran-normalized, or log of corrected counts (sctransform v2). Genes were marked differentially expressed if they exceeded an adjusted *p*-value threshold of 0.05.

We benchmarked our procedure’s ability to identify differentially expressed genes against log normalization and scran [46]. A Smart-seq3 PBMC dataset [29] and a PBMC Drop-seq dataset [69] were used as inputs to muscat [45]. Both the datasets were first annotated using the human PBMC reference from Azimuth (https://azimuth.hubmapconsortium.org/references/human_pbmc/) [70] and then only CD4 T, monocytes, and natural killer (NK) cells from each dataset were provided as inputs to muscat to generate a 3600 cells (nc=3600) dataset of 4000 (ng=4000) genes with three clusters (nk=3) and 5%, 10%, and 20% of differentially expressed genes in two conditions spread across three samples (ns=3). Muscat labels each gene as differentially or equivalently expressed across two samples within each cluster. We simulated three replicates for each scenario. For each normalization approach, differentially expressed genes between the two conditions were identified in each cluster. Differential expression (DE) analysis was performed using FindMarkers(logfc.threshold=0, min.pct=0) in Seurat using DESeq2, MAST, and Wilcoxon rank sum test. When calculating corrected counts for sctransform v2, we calculated the median sequencing depth across cells for both datasets, and set *n*_0_ to be the minimum of these two values. To compare the performance, we calculated the evaluation metrics using the iCOBRA package [71].

For the second analysis, we selected all NK cells (total 305) from the Smart-seq3 PBMC dataset and then downsampled them to have 20% of original sequencing depth using scuttle::downsampleMatrix(prop = 0.2, bycol = FALSE) [72]. We then identified differentially expressed genes using DESeq2, MAST, or Wilcoxon rank-sum test (adjusted *p*-value <0.05) across these two datasets, which are biologically identical, after processing the datasets using log-, scran-, sctransform v1-, and sctransform v2- based normalization. Additionally, we also performed a similar analysis but without simulated downsampling using HEK293 cells profiled using Quartz-Seq2 (167,199 median UMI) and Drop-seq (1,907 median UMI) by Mereu et al. [73]. Quartz-Seq2 dataset was randomly sampled to have an equal number of cells as the Drop-seq dataset (191 cells) before running differential expression tests to adjust for any compositional differences.

## Supplementary Information

**Additional file 1** Supplementary Figures and Tables.

**Additional file 2** Review history.

## Data Availability

The technical control datasets [8, 14], ChromiumV3 NIH3T3 cell line dataset and ChromiumV3 PBMC dataset [74] used in the main text are available publically from CaltechDATA repository record 1264 [75]. Other PBMC datasets are available from GSE132044 [69]. PBMC68k (“Fresh 68k PBMCs Donor A”, ChromiumV1) dataset is available from 10X Genomics website (https://www.10xgenomics.com/resources/datasets). HEK293 cell line datasets are available from GSE132044 [69] and GSE133549 [73]. Mouse cortex datasets are available from GSE132044 [69]. Smart-seq3 datasets are available from E-MTAB-8735 [29], Fetus dataset is available from GSE156793 [76], and the bone marrow dataset is available from GSE128639 [64]. Additionally, download public URLs for all datasets listed in Additional file 1: Table S1. Scripts to reproduce the analyses are available at: https://github.com/saketkc/scRNA_NB_comparison under a BSD 2-Clause license. Source code for sctransform along with the modifications described in this manuscript is available at: https://github.com/satijalab/sctransform. A Python implementation that interfaces with the Scanpy [77] package is available at: https://github.com/saketkc/pysctransform. All the source code and analysis scripts have also been made available in our Zenodo [78] Project (https://zenodo.org/record/5789958/) under a Creative Commons Attribution - 4.0 International license. To invoke ‘v2’ regularization in SCTransform using Seurat [70]: Analogously, to use SCTransform in Python (using Scanpy [77]):
