# Supplementary material for: Comparison and evaluation of statistical error models for scRNA-seq
Source: Genome Biol. 2022 Jan 18;23:27. doi: 10.1186/s13059-021-02584-9 (PMC8764781; doi:10.1186/s13059-021-02584-9)
Supplement: Supplementary file 1 — Additional file 1 Supplementary Figures and Tables. [file 13059_2021_2584_MOESM1_ESM.pdf]

Supplementary Information: Comparison and evaluation of statistical error models for scRNA-seq

Saket Choudhary[1] and Rahul Satija[1,2]

[1]New York Genome Center, 101 6th Ave, New York, NY, 10013 USA
[2]Center for Genomics and Systems Biology, New York University, 12 Waverly Pl, New York, NY, 10003 USA
{schoudhary,rsatija}@nygenome.org

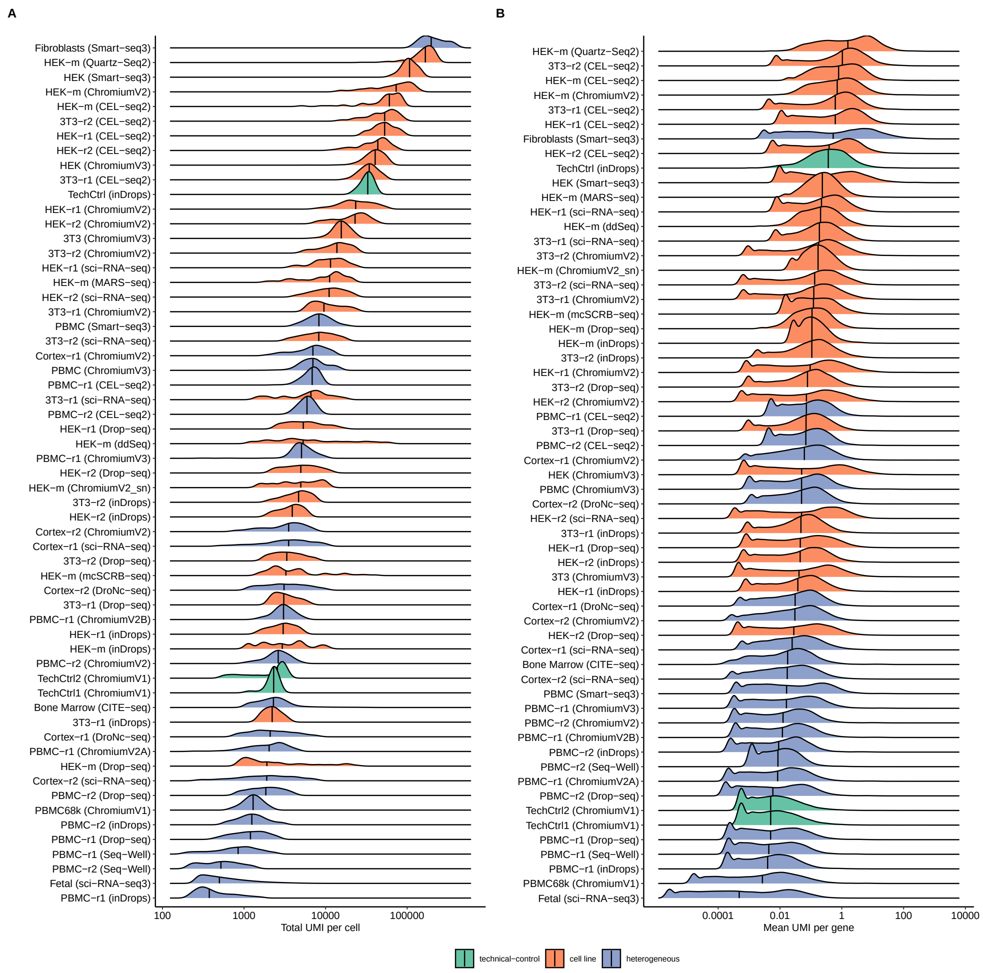

Figure S1: **UMI statistics for the 59 datasets analyzed in this manuscript. A)** Distribution of total UMI per cell across datasets **B)** Distribution of mean UMI per gene across datasets (technical control = endogeneous or spike-in RNA; cell line = HEK293 and 3T3 cell lines; heterogeneous = samples consisting of multiple cell types).

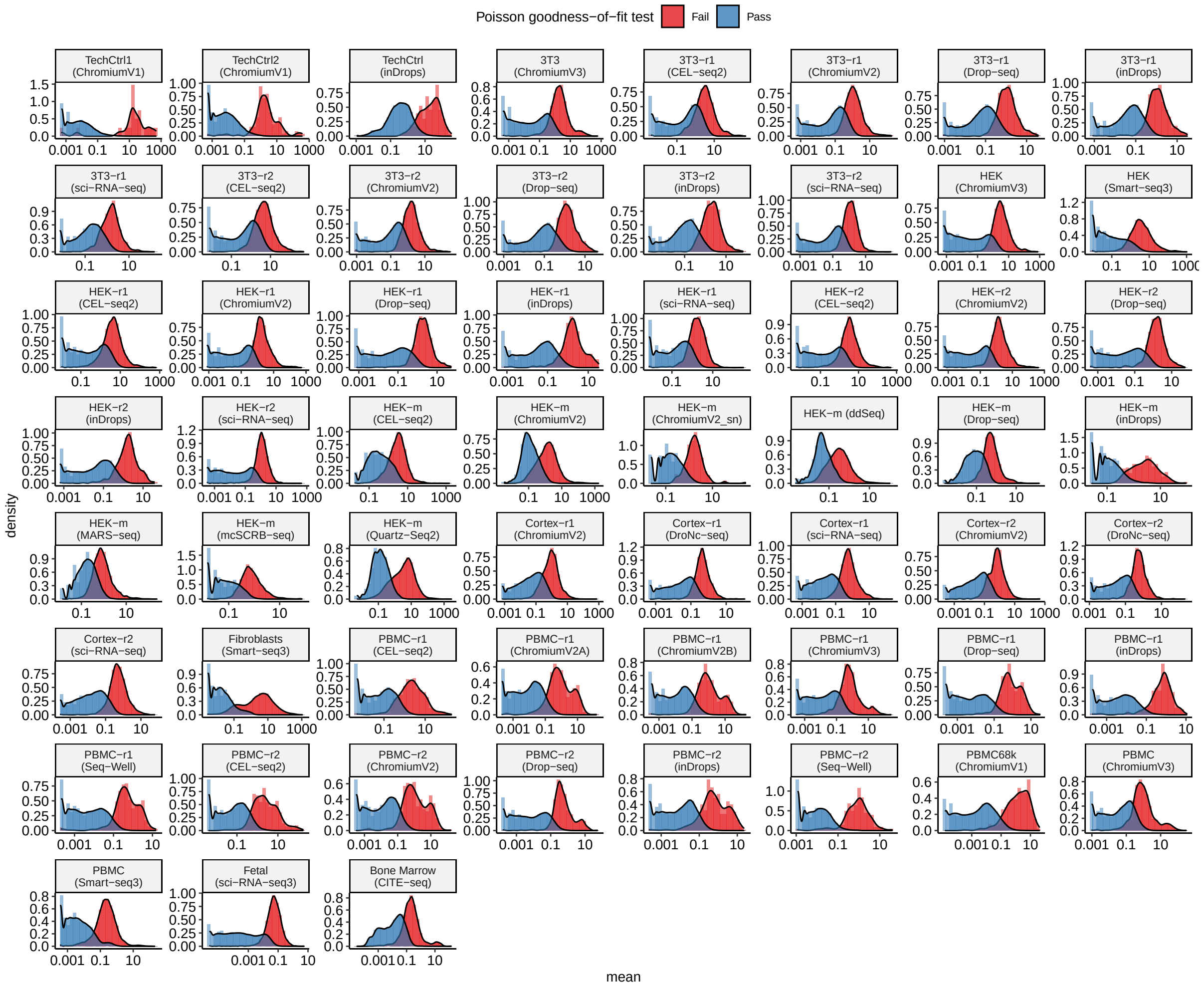

Figure S2: **Genes that exhibit Poisson heterogeneity are more lowly expressed.** In each dataset, we performed a per-gene goodness-of-fit test based on a GLM with a Poisson error model (Supplementary Methods). Shown are the distribution of gene abundances (average UMI/gene) for genes that passed (blue) and failed (red) the goodness-of-fit test.

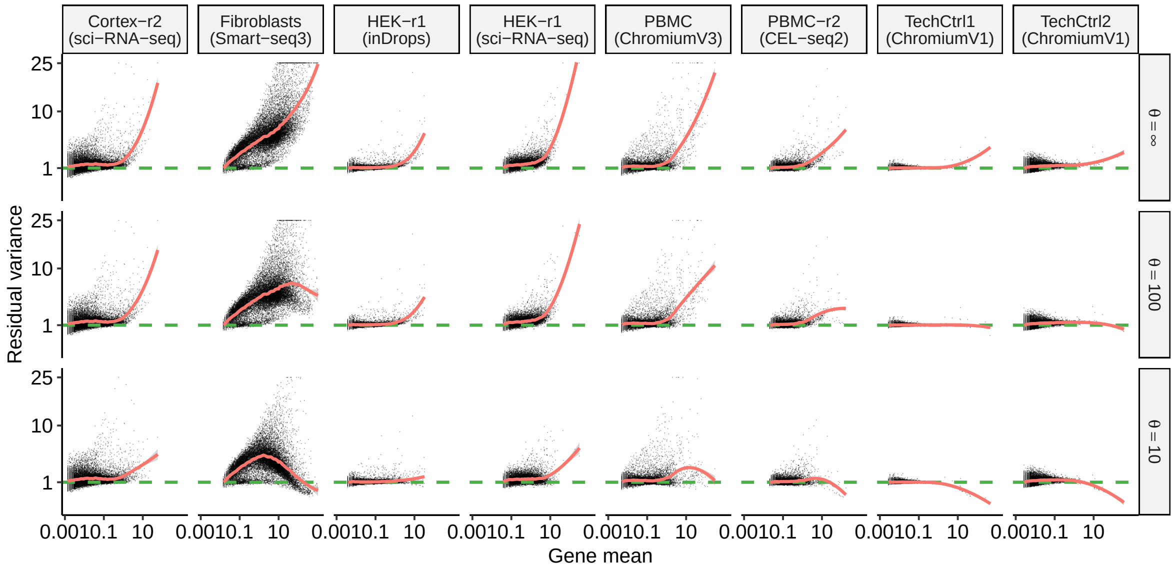

Figure S3: **Relationship between gene abundance and the variance of Pearson residuals**. Values shown are resulting from an NB GLM with three different values of θ. Same as Figure 2D but with per-gene estimates highlighted instead of smoothed curves.

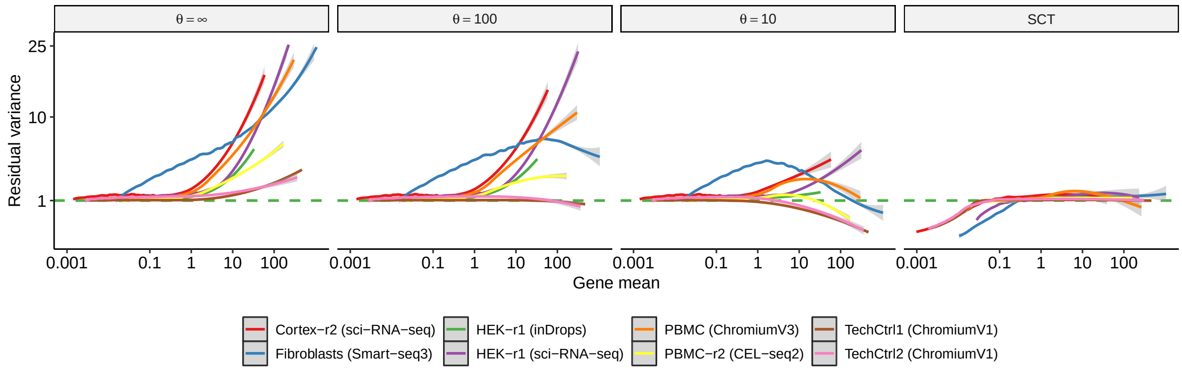

Figure S4: **Relationship between gene abundance and the variance of Pearson residuals**. Same as Figure 2D but additionally showing results for sctransform (v2 regularization).

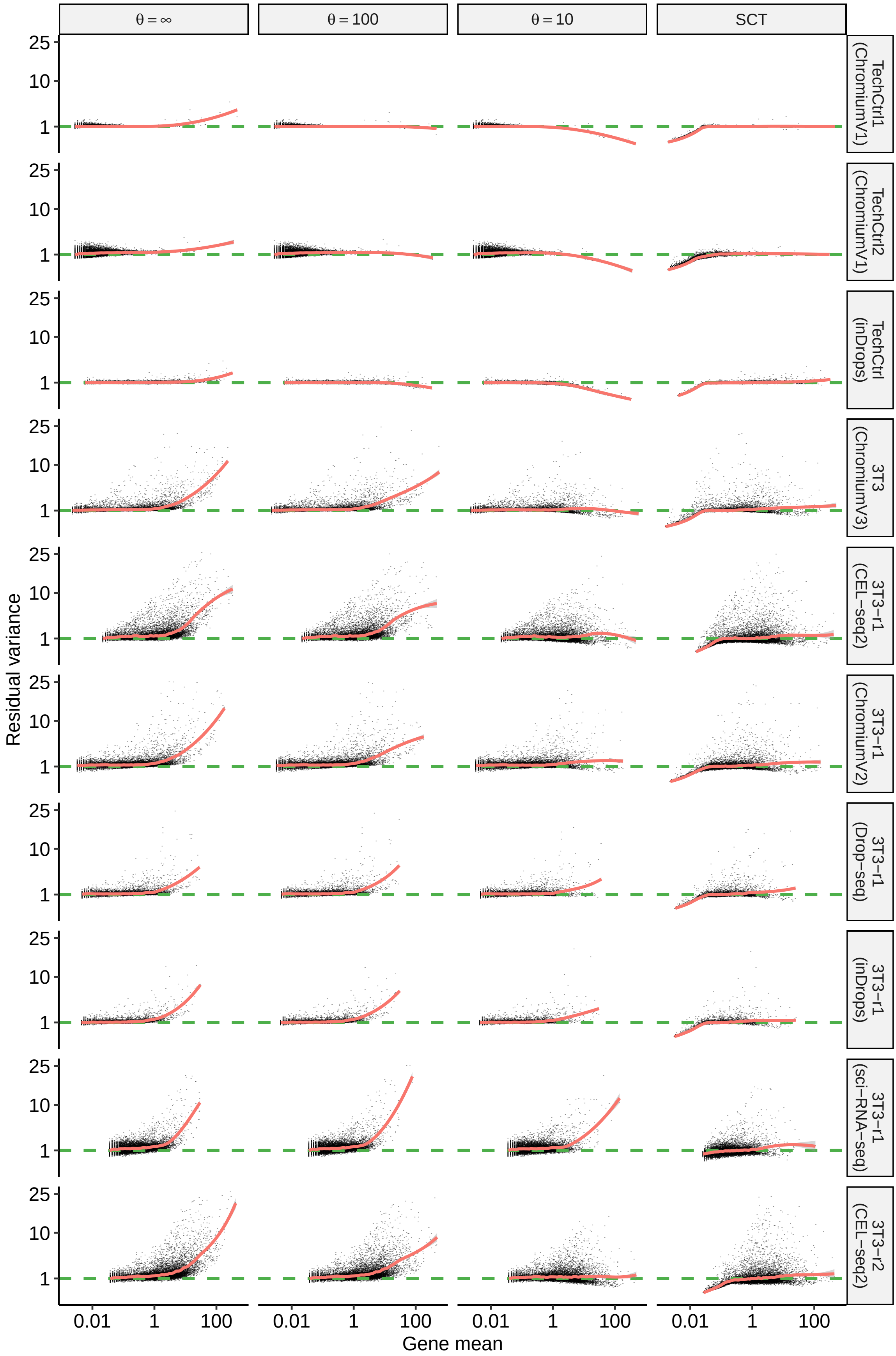

Figure S5: **Evaluating variance stabilization for different error models.** Same as in Figure 2C, but with additional datasets 1-10. Also shown are results from sctransform (v2 regularization).

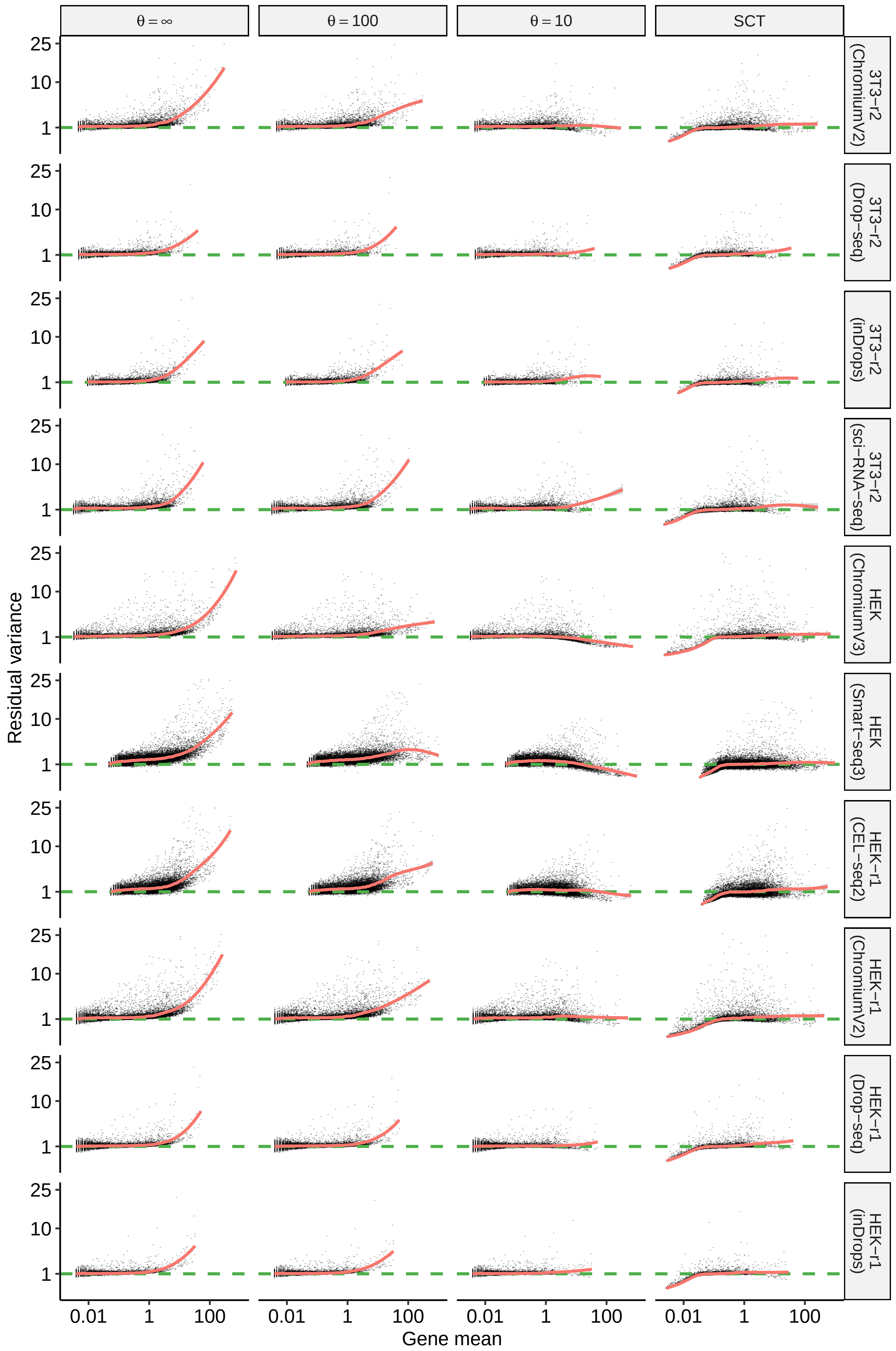

Figure S6: **Evaluating variance stabilization for different error models.** Same as in Figure 2C, but with additional datasets 11-20. Also shown are results from sctransform (v2 regularization).

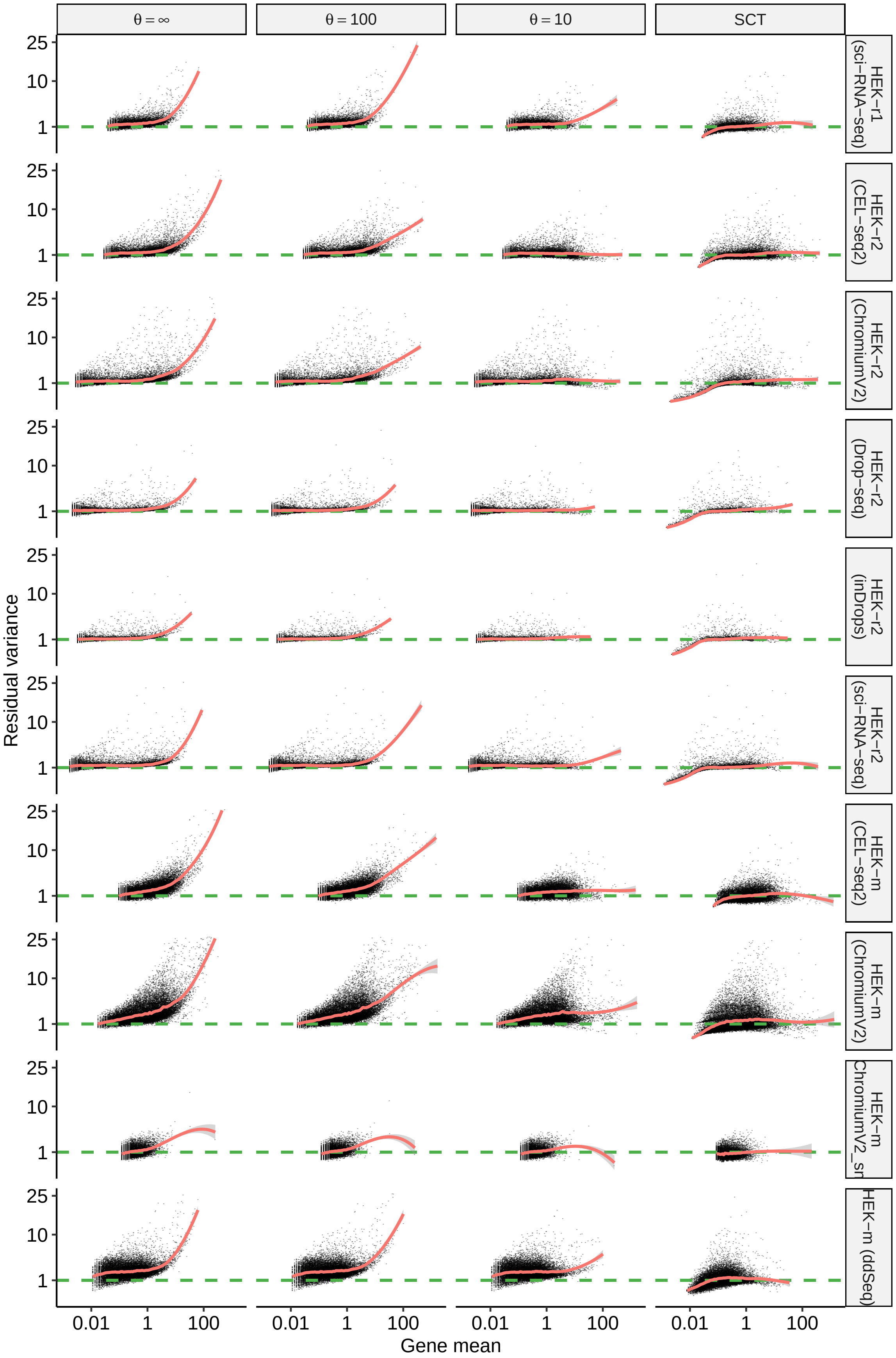

Figure S7: **Evaluating variance stabilization for different error models.** Same as in Figure 2C, but with additional datasets 21-30. Also shown are results from sctransform (v2 regularization).

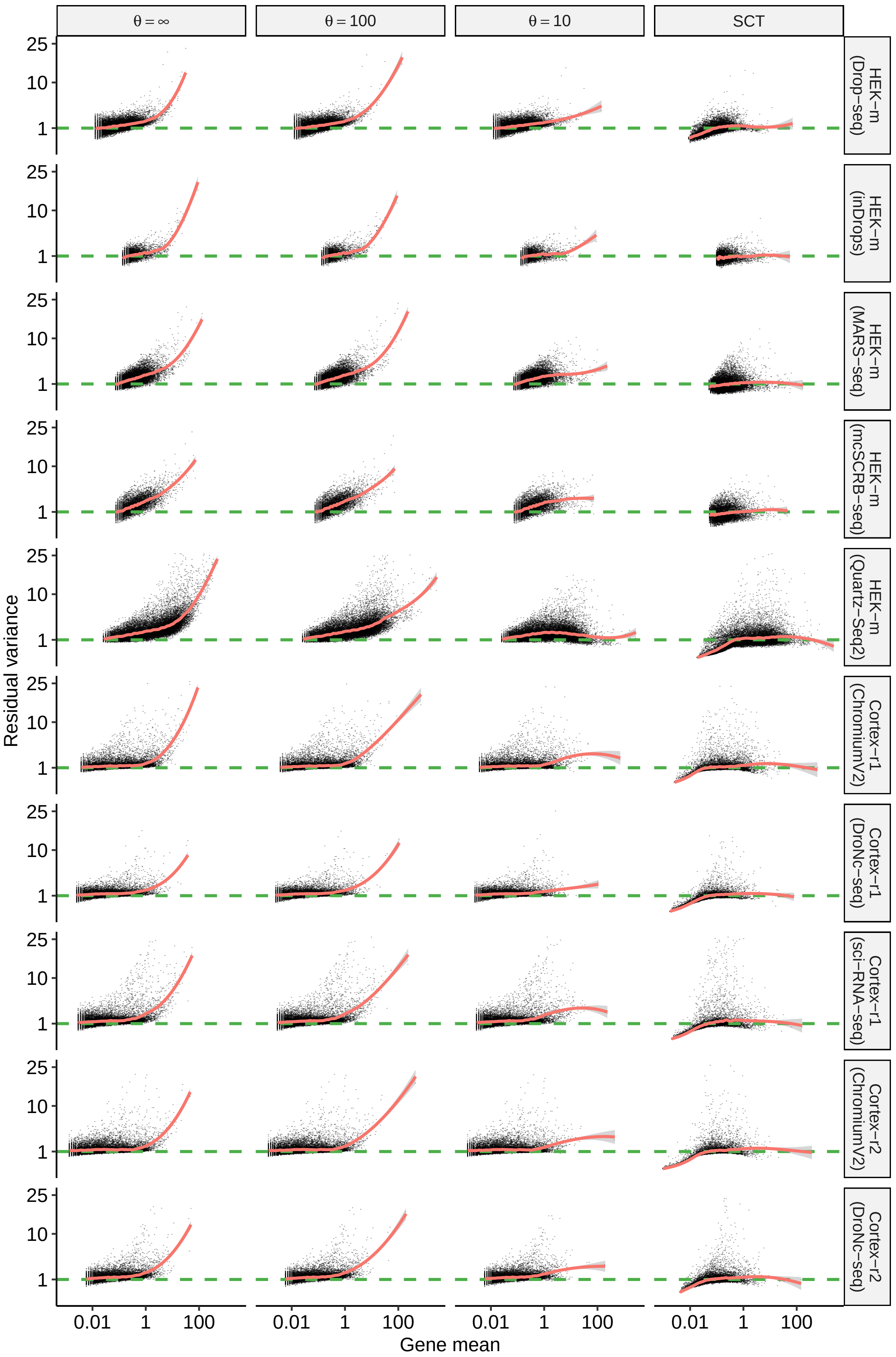

Figure S8: **Evaluating variance stabilization for different error models.** Same as in Figure 2C, but with additional datasets 31-40. Also shown are results from sctransform (v2 regularization).

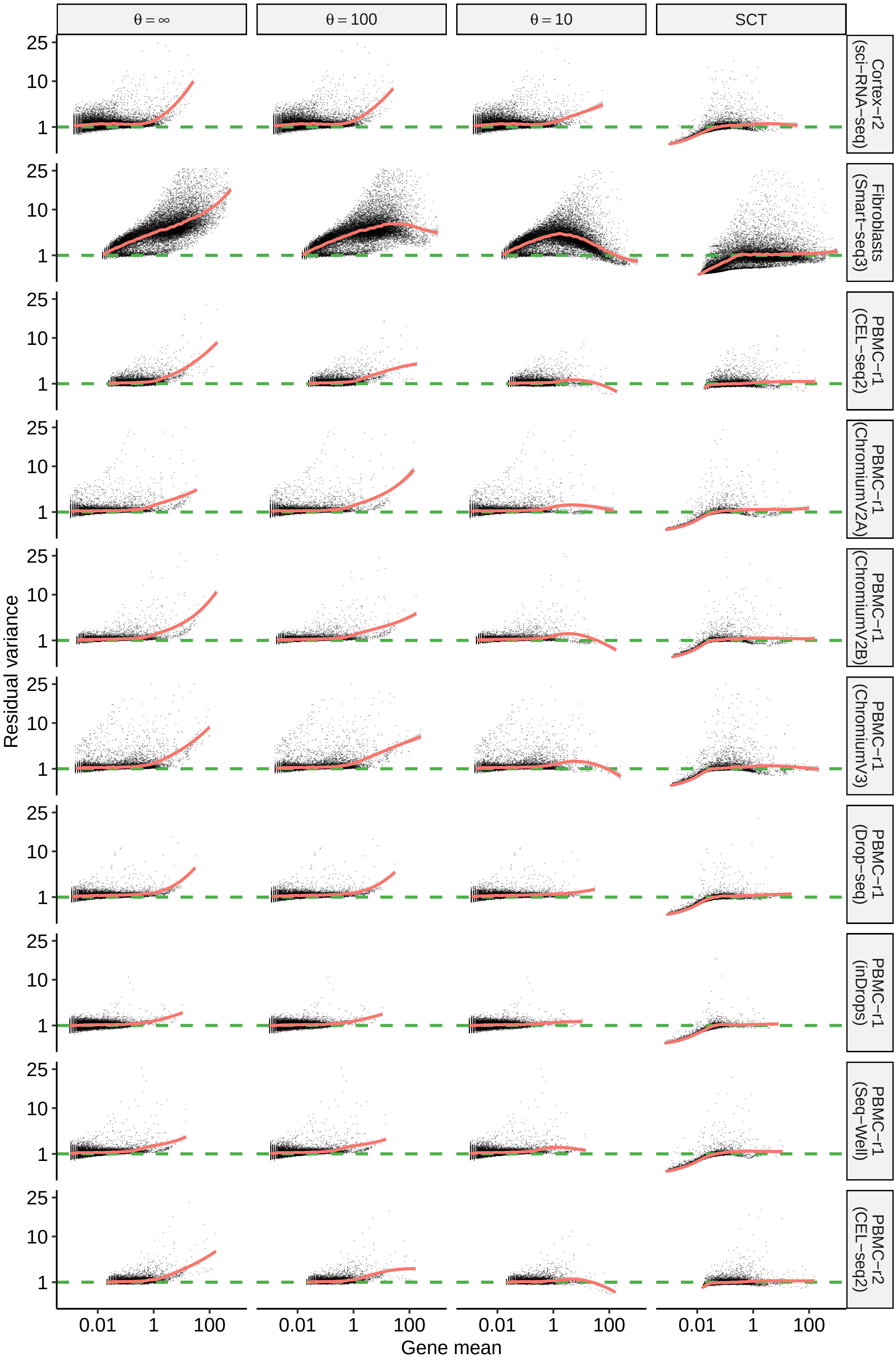

Figure S9: **Evaluating variance stabilization for different error models.** Same as in Figure 2C, but with additional datasets 41-50. Also shown are results from sctransform (v2 regularization).

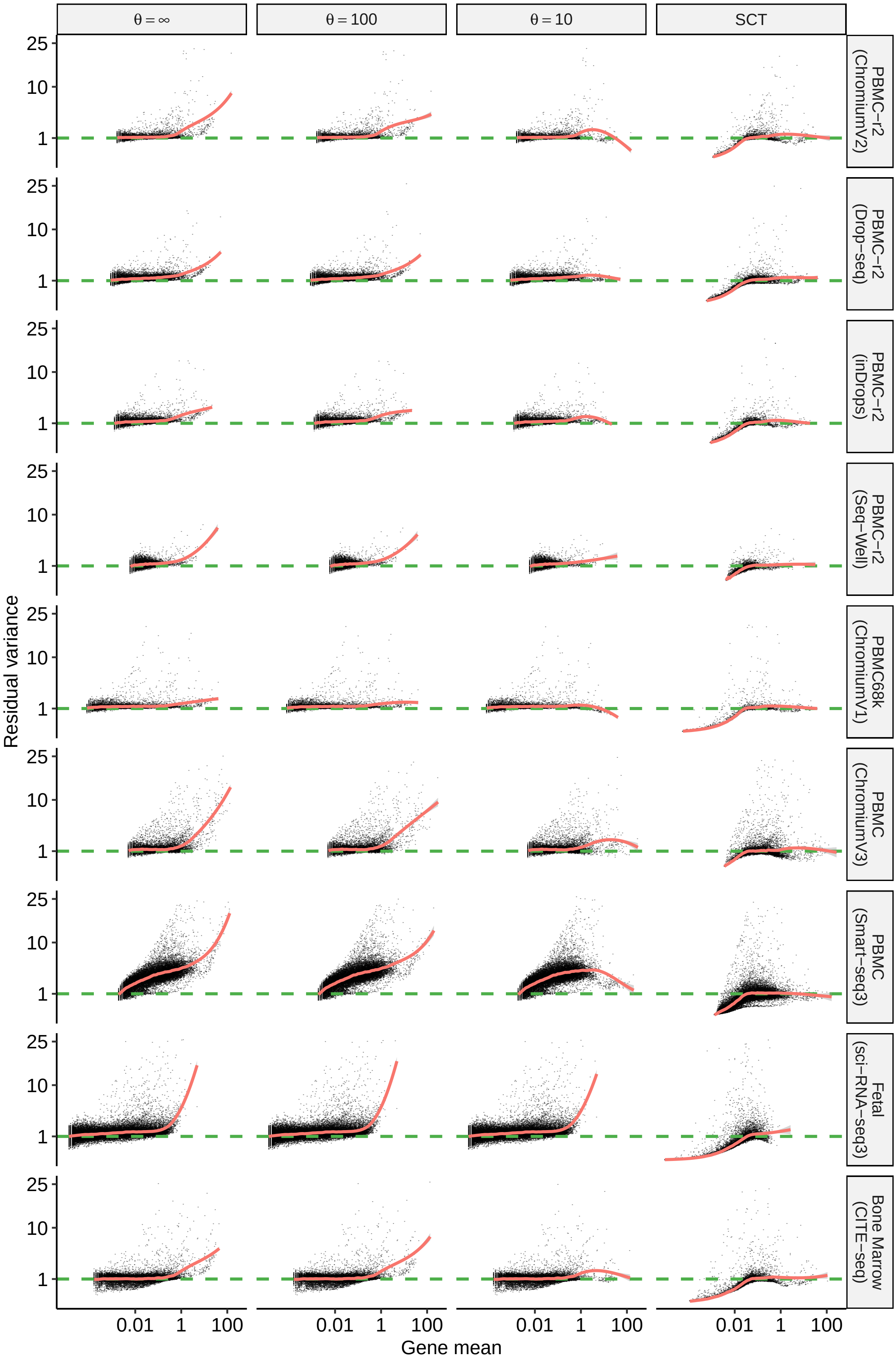

Figure S10: **Evaluating variance stabilization for different error models**. Same as in Figure 2C, but with additional datasets 51-59. Also shown are results from sctransform (v2 regularization).

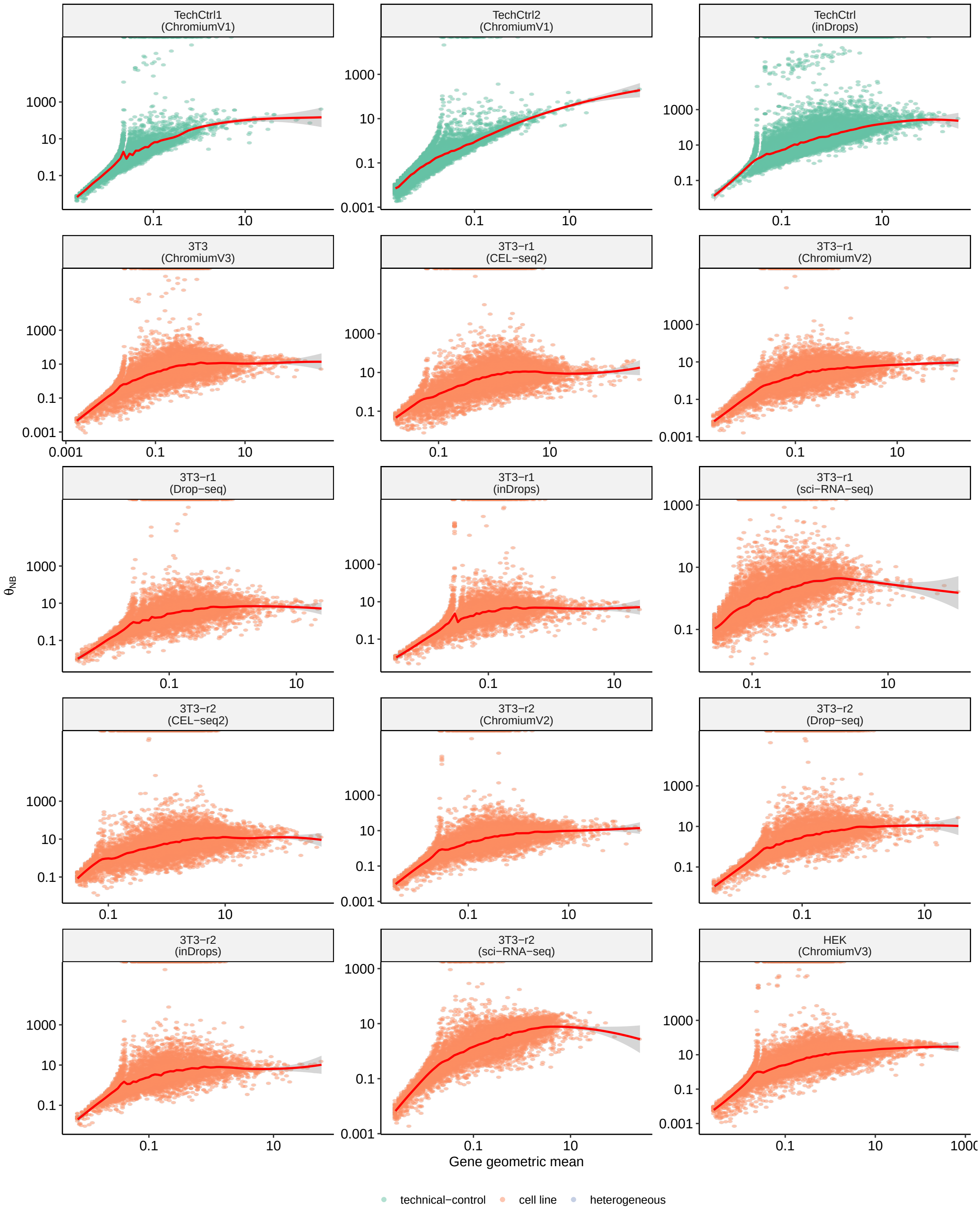

Figure S11: **Relationship between inverse overdispersion parameter θ and gene abundance μ.** Overdispersion was estimated using all cells after accounting for library size using a negative-binomial GLM. The red curve indicates a LOESS fit. All datasets exhibit a relationship between gene mean and θ [Datasets 1-15].

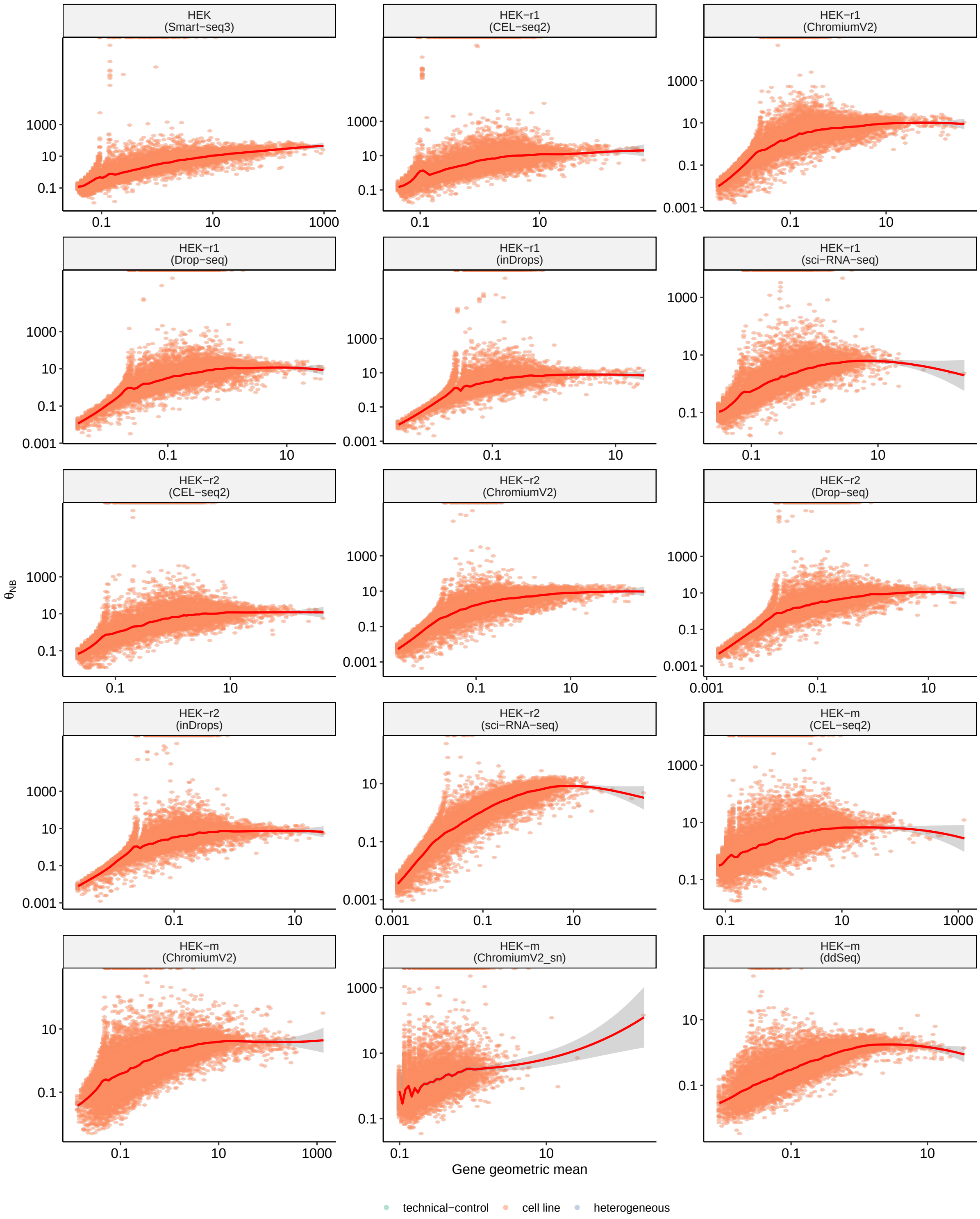

Figure S12: **Relationship between inverse overdispersion parameter θ and gene abundance μ.** Overdispersion was estimated using all cells after accounting for library size using a negative-binomial GLM. The red curve indicates a LOESS fit. All datasets exhibit a relationship between gene mean and θ [Datasets 16-30].

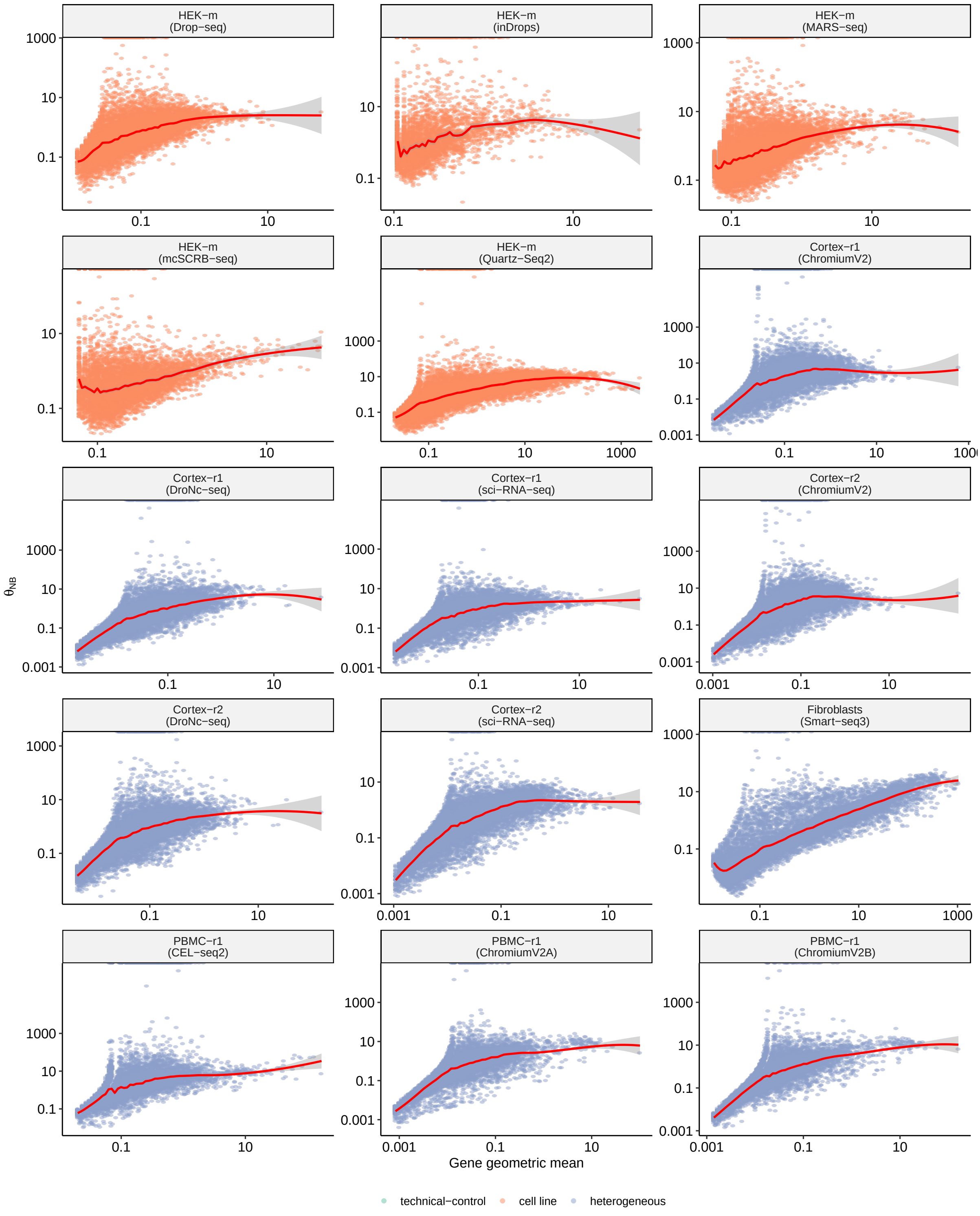

Figure S13: **Relationship between inverse overdispersion parameter θ and gene abundance μ.** Overdispersion was estimated using all cells after accounting for library size using a negative-binomial GLM. The red curve indicates a LOESS fit. All datasets exhibit a relationship between gene mean and θ [Datasets 31-45].

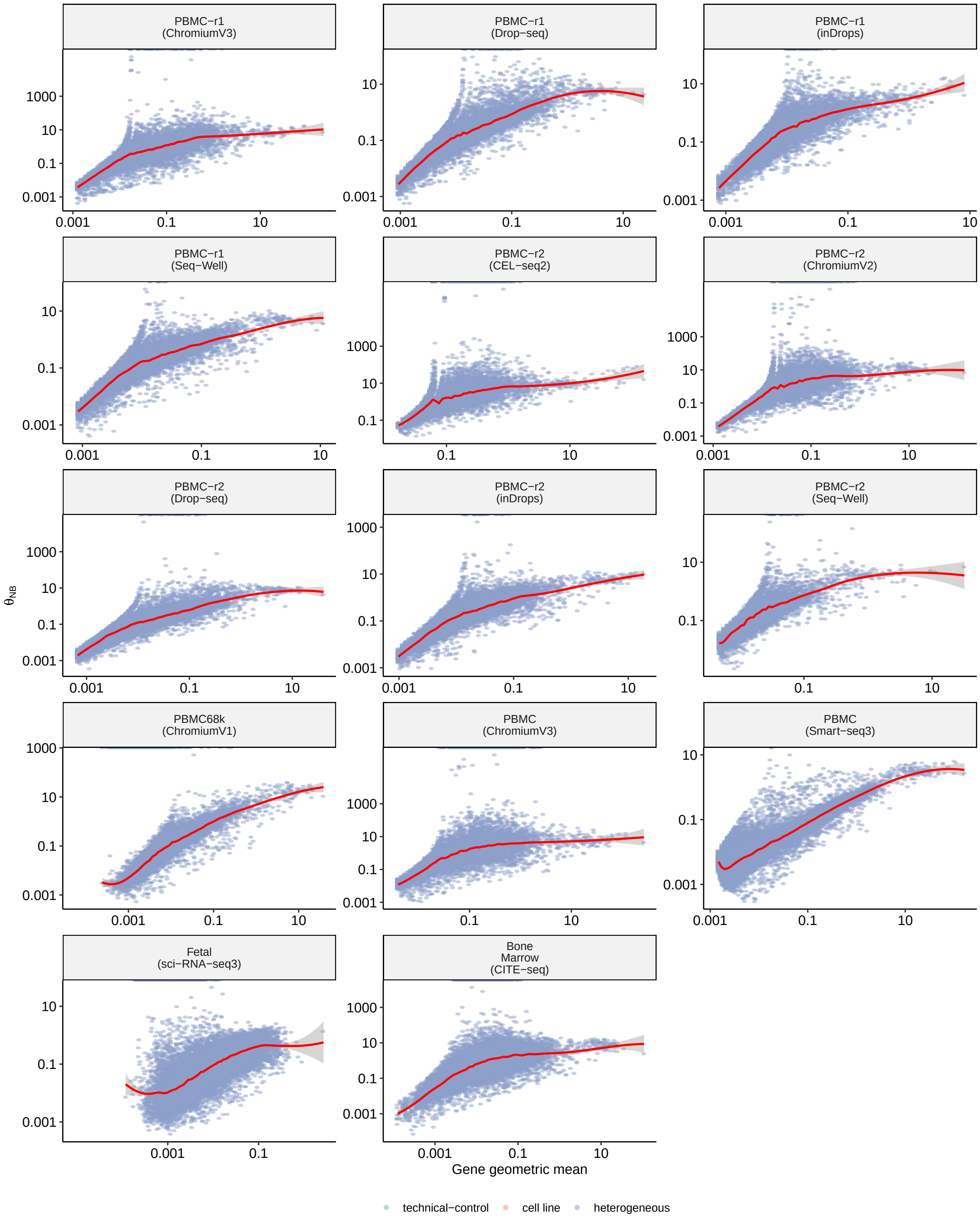

Figure S14: **Relationship between inverse overdispersion parameter θ and gene abundance μ.** Overdispersion was estimated using all cells after accounting for library size using a negative-binomial GLM. The red curve indicates a LOESS fit. All datasets exhibit a relationship between gene mean and θ [Datasets 46-59].

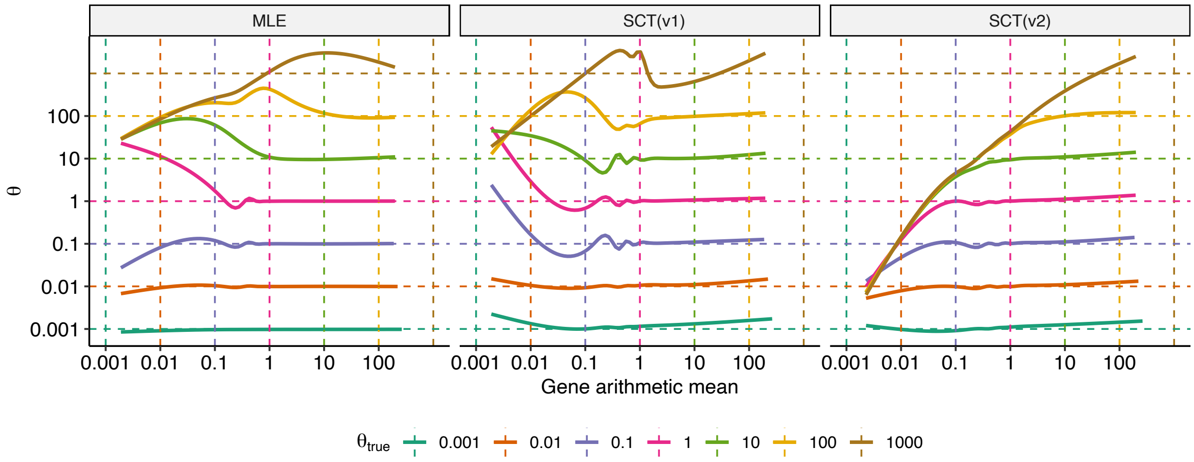

Figure S15: **Estimation of dispersion in simulated datasets.** Using mean counts distribution from PBMCs profiled using Smart-seq3, synthetic counts matrices were generated using a fixed $\theta = \{0.001, 0.01, 0.1, 1, 10, 100\}$. There is a bias in estimated θ from all the three methods: MLE (glmGamPoi (1)), SCT (sctransform) and SCT2 (sctransform, v2 regularization). The bias arises from difficulty in estimating the true θ when $\mu < 1$ and $\mu < \theta$. We note that the variance of the NB model is given by $\mu_{gc} + \mu_{gc}^2/\theta_g$. The second term approaches 0 for small values of μ, which is where we observe this bias. Therefore, the bias in parameter estimation has minimal impact on both the expected NB variance, and the final Pearson residuals (2).

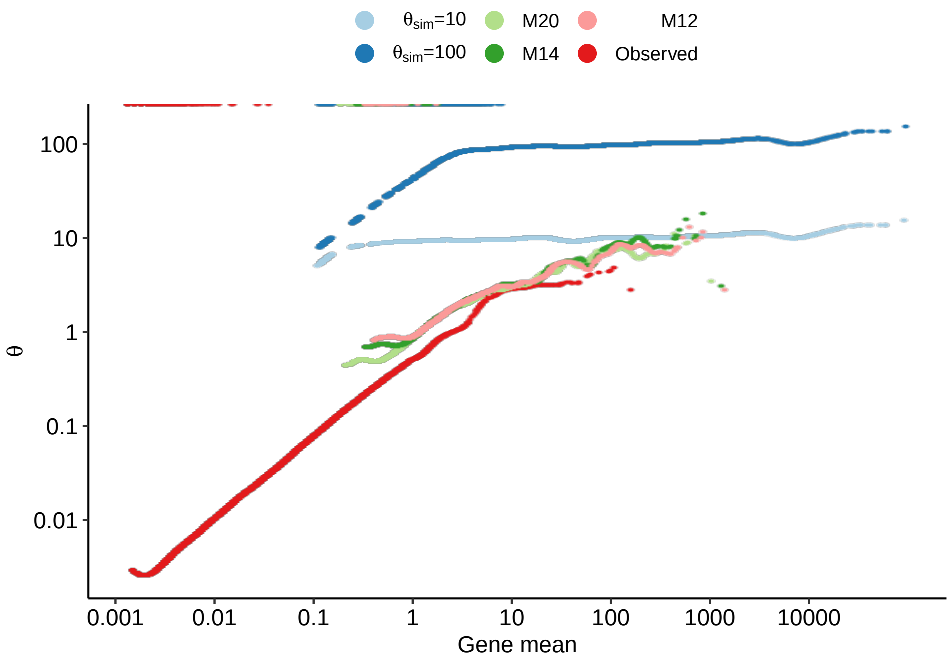

Figure S16: **Effect of 'upsampling' on $\mu - \theta$ relationship** Relationship between gene mean and dispersion observed in PBMC Smart-seq3 dataset, simulated dataset with different true dispersions ($\theta_{\text{sim}} = 10$ and 100) and Metacells (M20, M14, M12). The simulated datasets were generated using mean counts from the observed PBMC Smart-seq3 dataset, but by 'upsampling' the means to be 500 times larger. Metacells were generated using MetaCell (3) using different parameters of K for the KNN graph. M20, M14, and M12 represents 20, 14, and 12 metacells constructed using K = 200, 300, and 400 respectively.

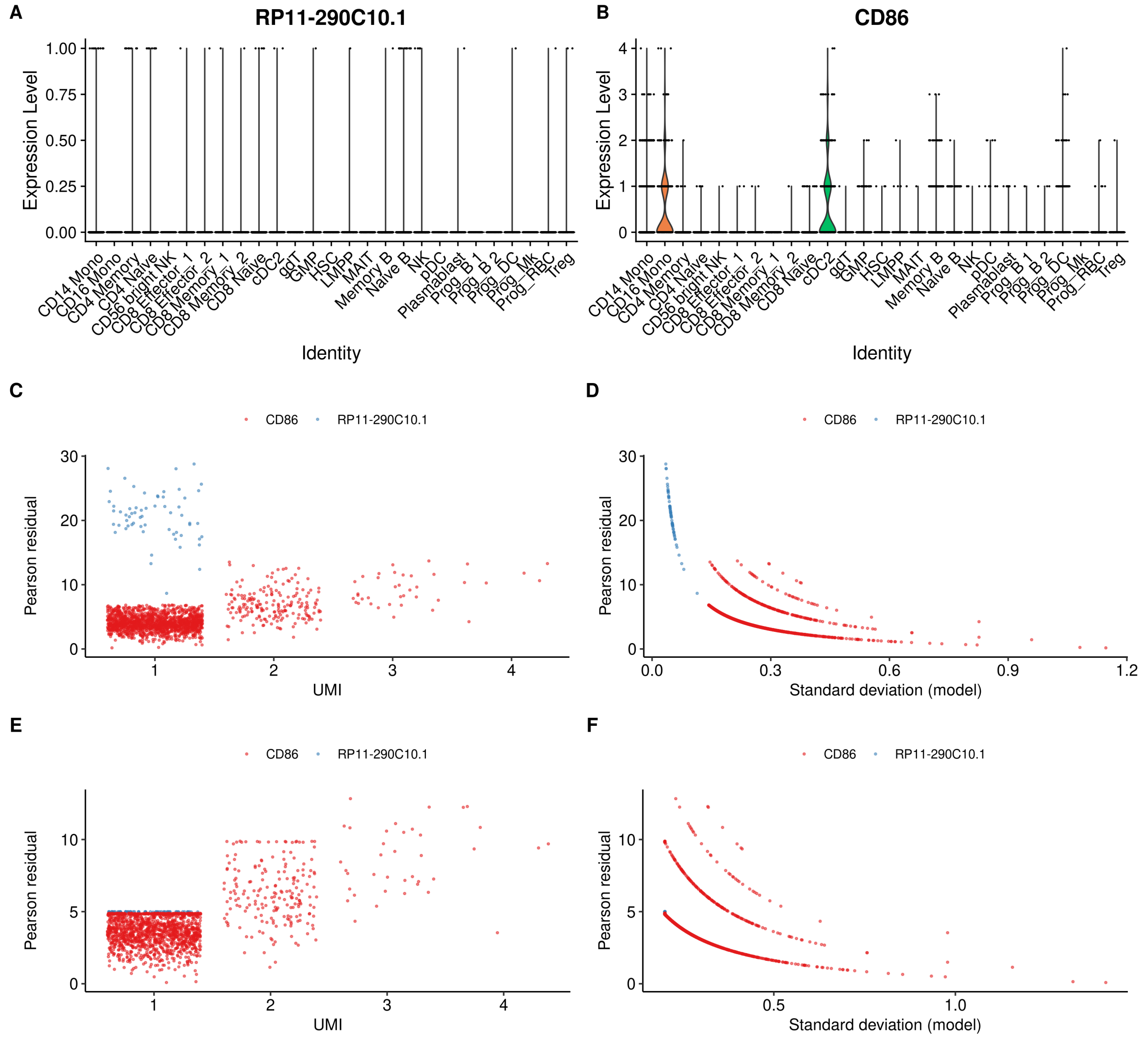

Figure S17: **Impact of thresholding minimum variance on variable feature selection.**. **A)** Distribution of UMIs (Y-axis) across celltypes for RP11-290C10.1 (non-variable gene). **B)** Distribution and UMIs across celltypes for CD86 (variable marker gene). **C)** Distribution of Pearson residual calculated using sctransform v1 with respect to model-estimated standard deviation. **D)** Relationship between model-estimated standard deviation and resulting Pearson residual calculated using sctransform v1. For RP11-290C10.1, sctransform v1 calculates very high Pearson residuals for cells with 1 UMI due to a very low estimated standard-deviation. **E) and F)** Analogous to **C and D** with estimates from sctransform (v2 regularization). Celltypes in **A-B** are from the original dataset (4).

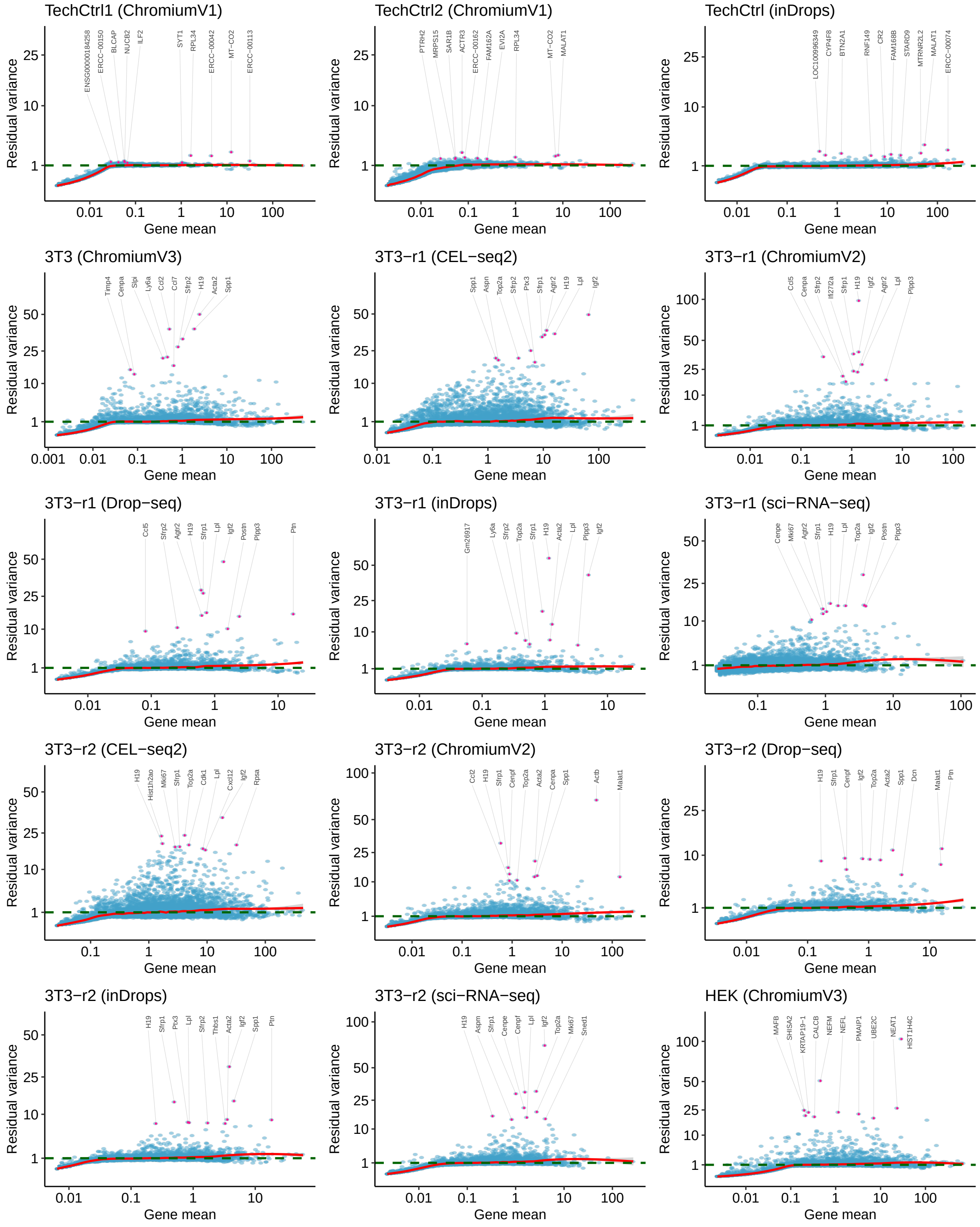

Figure S18: **Variance stabilization achieved by sctransform2 across datasets**. Y-axis shows variation of Pearson residuals as estimated by sctransform (v2 regularization) for datasets 1-15. Top 10 genes with the highest residual variances are highlighted.

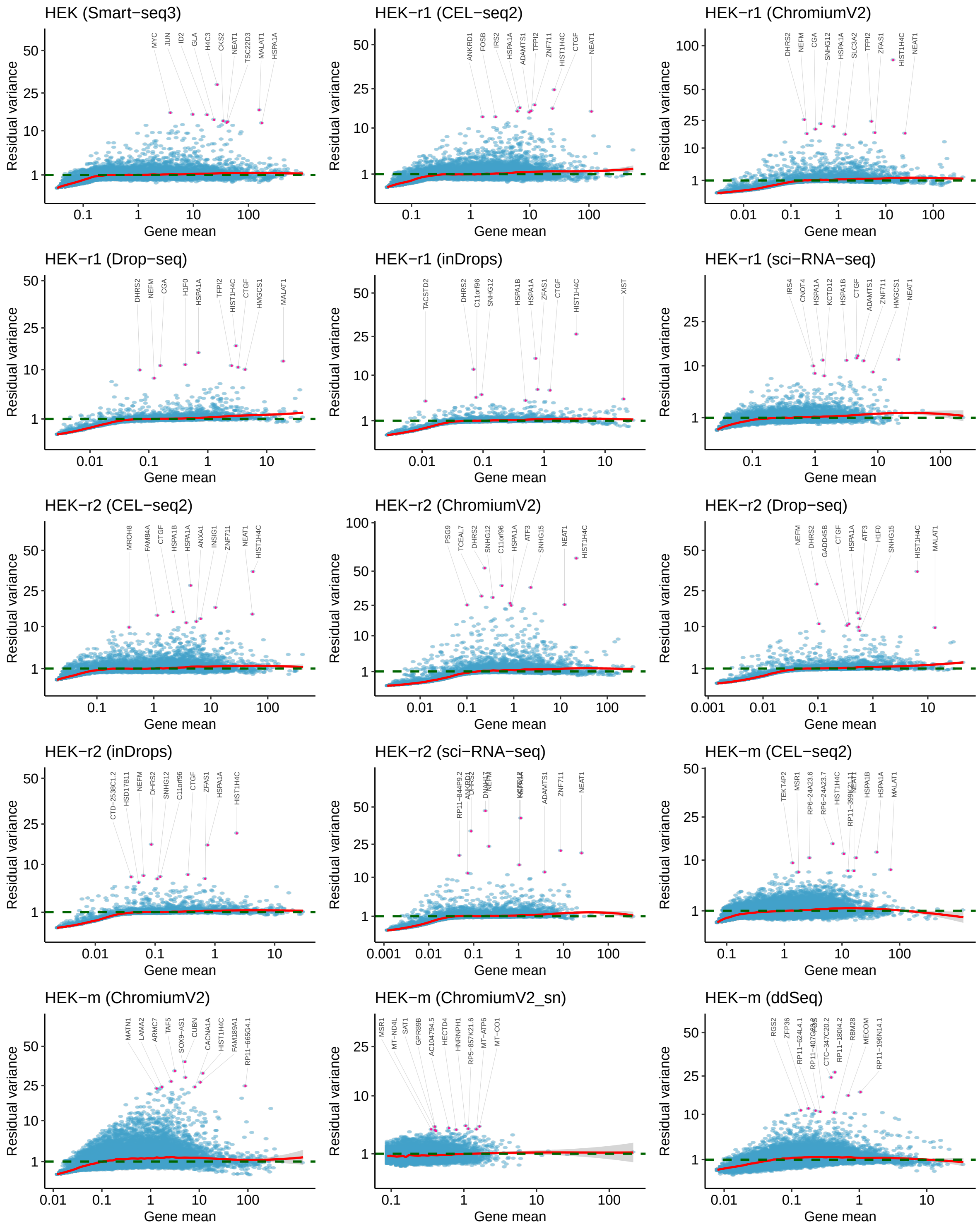

Figure S19: **Variance stabilization achieved by sctransform2 across datasets**. Y-axis shows variation of Pearson residuals as estimated by sctransform (v2 regularization) for datasets 16-30. Top 10 genes with the highest residual variances are highlighted.

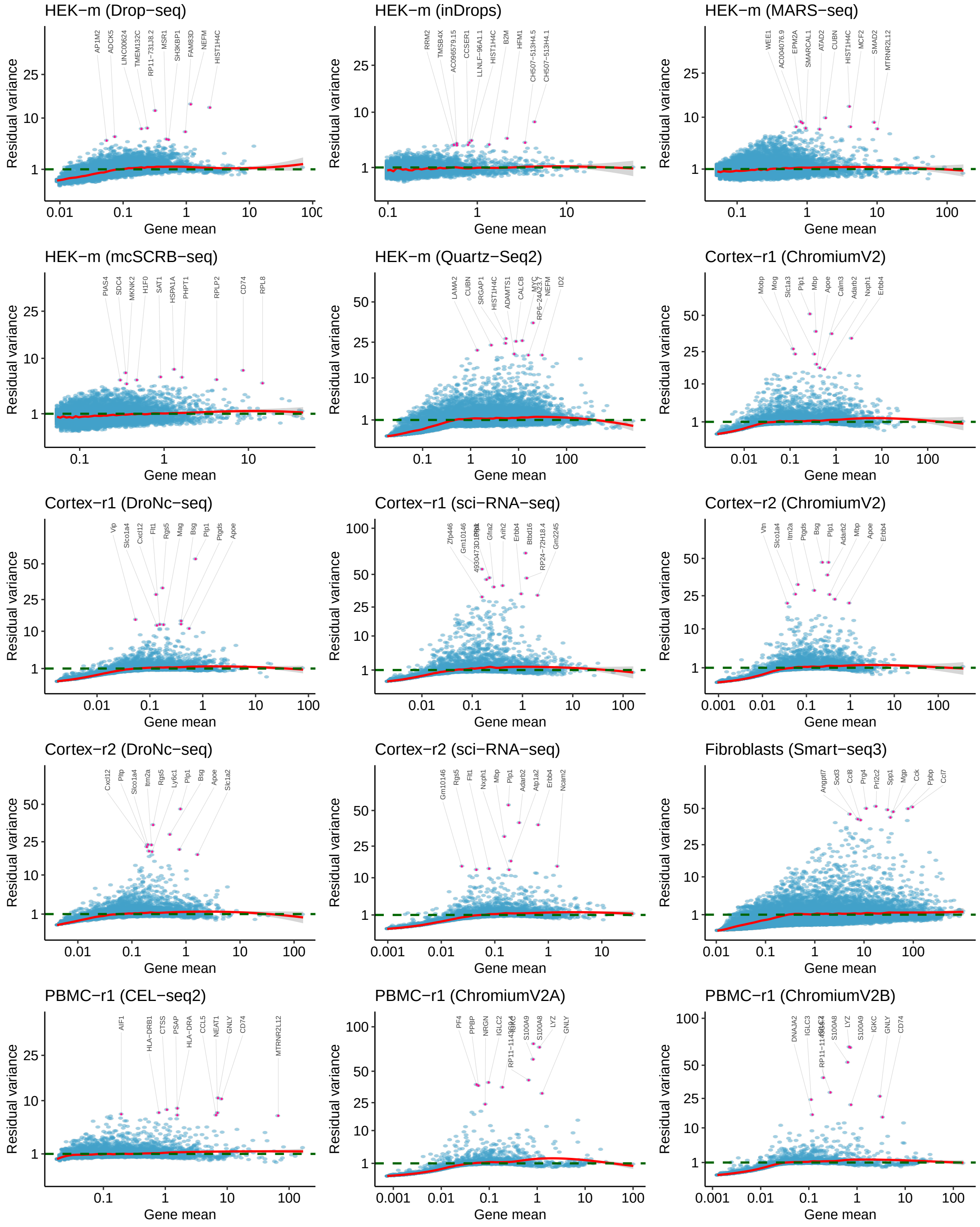

Figure S20: **Variance stabilization achieved by sctransform2 across datasets**. Y-axis shows variation of Pearson residuals as estimated by sctransform (v2 regularization) for datasets 31-45. Top 10 genes with the highest residual variances are highlighted.

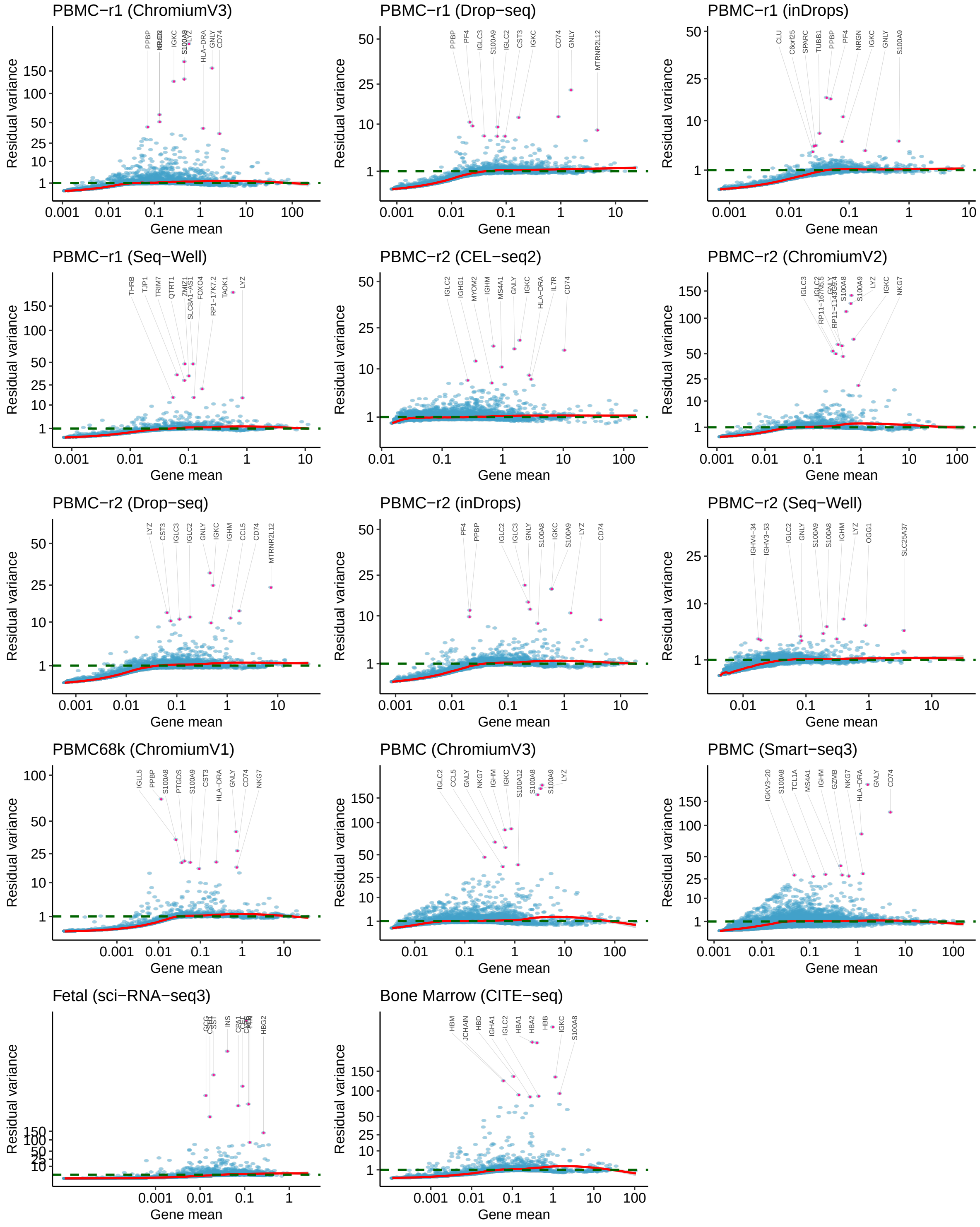

Figure S21: **Variance stabilization achieved by sctransform2 across datasets**. Y-axis shows variation of Pearson residuals as estimated by sctransform (v2 regularization) for datasets 46-59. Top 10 genes with the highest residual variances are highlighted.

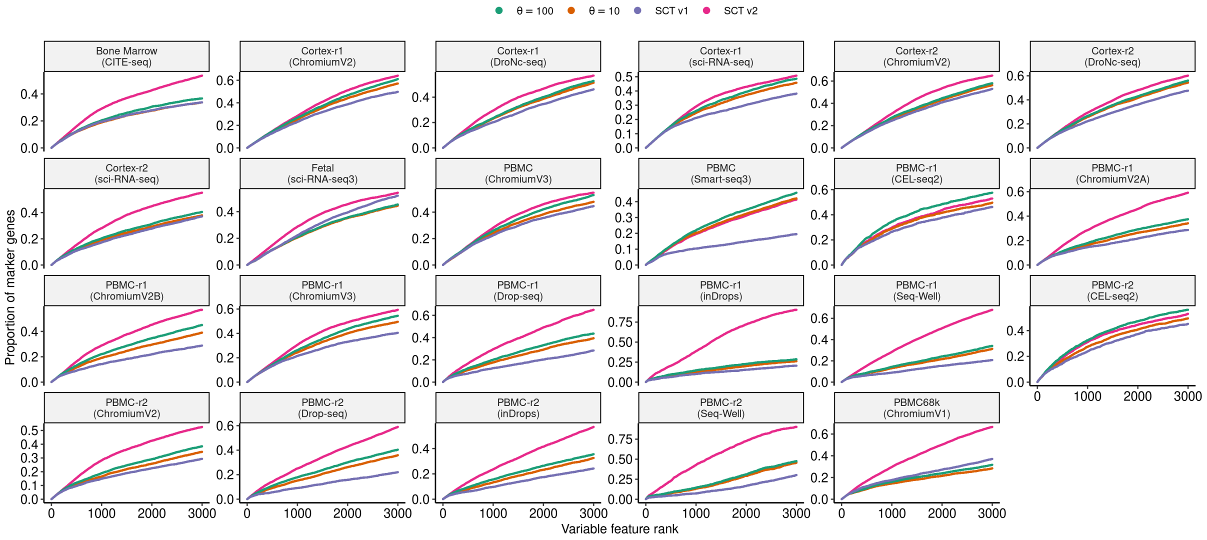

Figure S22: **Overlap of variable genes and marker genes.** X-axis represents variable genes ordered by decreasing residual variance for $\theta = \{100, 10\}$, sctransform v1, and sctransform v2. Y-axis shows cumulative proportion overlap with marker genes. Marker genes were identified by unsupervised clustering on log-normalized data. Number of features and proportion overlap are provided in Supplementary Tables S3 and S4.

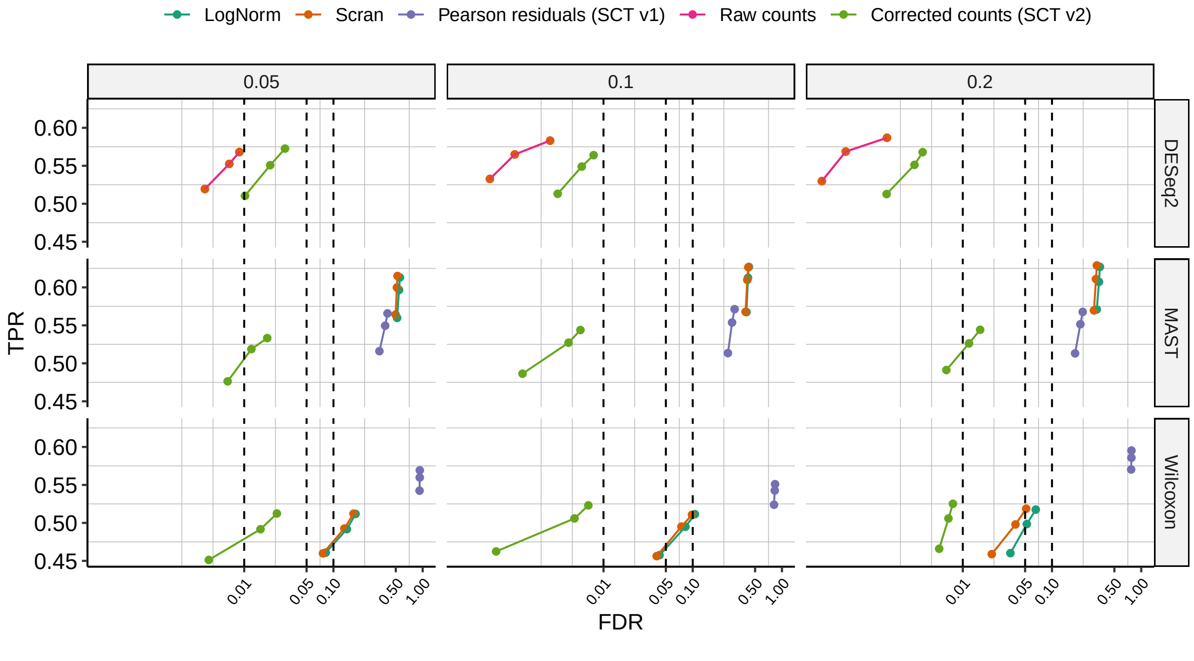

Figure S23: **Benchmarking differential expression analysis.** Observed overall true positive rate (TPR) and false discovery rate (FDR) values for DE genes at FDR cutoffs of 1%, 5%, and 10% using a DESeq2 (5), MAST (6) and Wilcoxon (7) rank sum test. Dashed vertical lines indicate desired FDRs. Methods that control FDR at their desired level should fall to the left of the corresponding dashed line. Performances were averaged across three simulation replicates. Data was simulated with muscat (8) using three annotated cell types (CD4 T, monocytes and natural killer cells) from a Smart-seq3 and a Drop-seq PBMC dataset. Column titles indicate simulated proportion of DE genes while row titles indicate the DE method. DESeq2 was run using raw counts or sctransform v2 corrected counts as input. MAST and Wilocoxon test were run on log-normalized, scran-normalized, pearson residuals (SCT v1) or log of corrected counts (SCT v2).

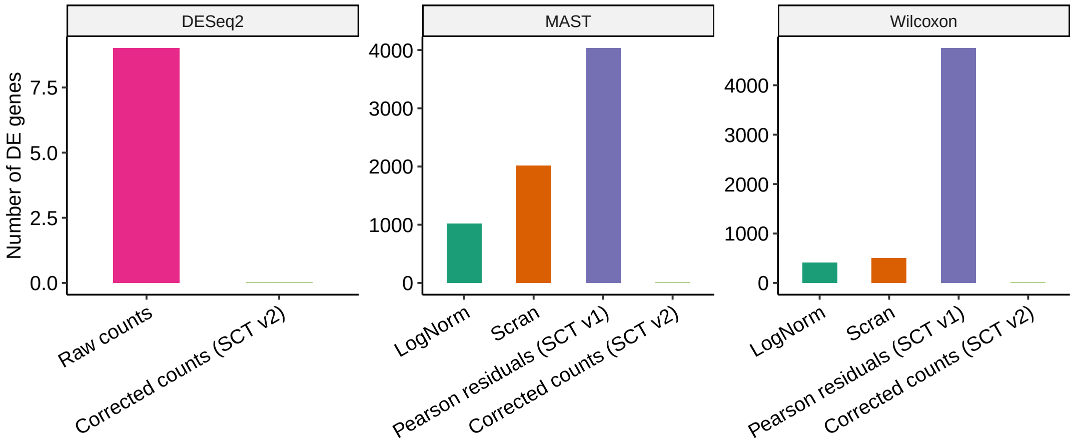

Figure S24: **Benchmarking differential expression analysis.** Number of differential expression genes identified between two groups of biological identical NK cells (PBMC Smart-seq3) where one group was randomly downsampled to 20% sequencing depth. Column titles show the DE testing method.

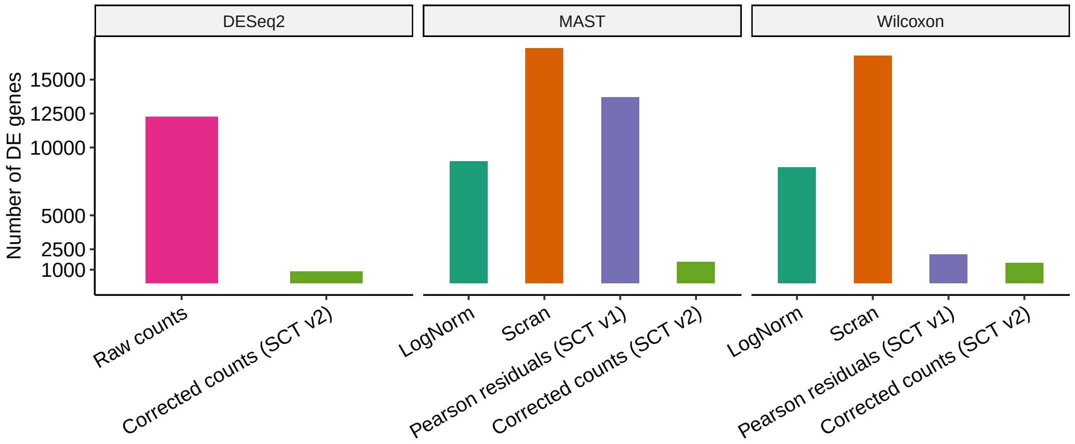

Figure S25: **Benchmarking differential expression analysis.** Number of differential expression genes identified between two groups of biological identical HEK293 cells profiled using Quartz-Seq2 and Drop-seq. Datasets were obtained from Mereu et al. (9). Column titles show the DE testing method.

Sample name	Technology	Tissue	Datatype	Raw data	Reference
TechnicalControl1 (ChromiumV1)	ChromiumV1	TechnicalControl	technical-control	link	(10, 11)
TechnicalControl2 (ChromiumV1)	ChromiumV1	TechnicalControl	technical-control	link	(10, 11)
TechnicalControl (inDrops)	inDrops	TechnicalControl	technical-control	link	(11, 12)
3T3 (ChromiumV3)	ChromiumV3	3T3	cell line	link	(11)
3T3-r1 (ChromiumV2)	ChromiumV2	3T3	cell line	link	(13)
3T3-r1 (Drop-seq)	Drop-seq	3T3	cell line	link	(13)
3T3-r1 (inDrops)	inDrops	3T3	cell line	link	(13)
3T3-r1 (sci-RNA-seq)	sci-RNA-seq	3T3	cell line	link	(13)
3T3-r2 (CEL-seq2)	CEL-seq2	3T3	cell line	link	(13)
3T3-r2 (ChromiumV2)	ChromiumV2	3T3	cell line	link	(13)
3T3-r2 (Drop-seq)	Drop-seq	3T3	cell line	link	(13)
3T3-r2 (inDrops)	inDrops	3T3	cell line	link	(13)
3T3-r2 (sci-RNA-seq)	sci-RNA-seq	3T3	cell line	link	(13)
HEK (ChromiumV3)	ChromiumV3	HEK	cell line	link	(11)
HEK (Smart-seq3)	Smart-seq3	HEK	cell line	link	(14)
HEK-r1 (CEL-seq2)	CEL-seq2	HEK	cell line	link	(13)
HEK-r1 (ChromiumV2)	ChromiumV2	HEK	cell line	link	(13)
HEK-r1 (Drop-seq)	Drop-seq	HEK	cell line	link	(13)
HEK-r1 (inDrops)	inDrops	HEK	cell line	link	(13)
HEK-r1 (sci-RNA-seq)	sci-RNA-seq	HEK	cell line	link	(13)
HEK-r2 (CEL-seq2)	CEL-seq2	HEK	cell line	link	(13)
HEK-r2 (ChromiumV2)	ChromiumV2	HEK	cell line	link	(13)
HEK-r2 (Drop-seq)	Drop-seq	HEK	cell line	link	(13)
HEK-r2 (inDrops)	inDrops	HEK	cell line	link	(13)
HEK-r2 (sci-RNA-seq)	sci-RNA-seq	HEK	cell line	link	(13)
HEK-m (CEL-seq2)	CEL-seq2	HEK	cell line	link	(9)
HEK-m (ChromiumV2)	ChromiumV2	HEK	cell line	link	(9)
HEK-m (ChromiumV2_sn)	ChromiumV2	HEK	cell line	link	(9)
HEK-m (ddSeq)	ddSeq	HEK	cell line	link	(9)
HEK-m (Drop-seq)	Drop-seq	HEK	cell line	link	(9)
HEK-m (inDrops)	inDrops	HEK	cell line	link	(9)
HEK-m (MARS-seq)	MARS-seq	HEK	cell line	link	(9)
HEK-m (mcSCRB-seq)	mcSCRB-seq	HEK	cell line	link	(9)
HEK-m (Quartz-Seq2)	Quartz-Seq2	HEK	cell line	link	(9)
Cortex-r1 (ChromiumV2)	ChromiumV2	Cortex	heterogeneous	link	(13)
Cortex-r1 (DroNc-seq)	DroNc-seq	Cortex	heterogeneous	link	(13)
Cortex-r1 (sci-RNA-seq)	sci-RNA-seq	Cortex	heterogeneous	link	(13)
Cortex-r2 (ChromiumV2)	ChromiumV2	Cortex	heterogeneous	link	(13)
Cortex-r2 (DroNc-seq)	DroNc-seq	Cortex	heterogeneous	link	(13)
Cortex-r2 (sci-RNA-seq)	sci-RNA-seq	Cortex	heterogeneous	link	(13)
Fibroblasts (Smart-seq3)	Smart-seq3	Fibroblasts	heterogeneous	link	(14)
PBMC-r1 (CEL-seq2)	CEL-seq2	PBMC	heterogeneous	link	(13)
PBMC-r1 (ChromiumV2A)	ChromiumV2	PBMC	heterogeneous	link	(13)
PBMC-r1 (ChromiumV2B)	ChromiumV2	PBMC	heterogeneous	link	(13)
PBMC-r1 (ChromiumV3)	ChromiumV3	PBMC	heterogeneous	link	(13)
PBMC-r1 (Drop-seq)	Drop-seq	PBMC	heterogeneous	link	(13)
PBMC-r1 (inDrops)	inDrops	PBMC	heterogeneous	link	(13)
PBMC-r1 (Seq-Well)	Seq-Well	PBMC	heterogeneous	link	(13)
PBMC-r2 (CEL-seq2)	CEL-seq2	PBMC	heterogeneous	link	(13)
PBMC-r2 (ChromiumV2)	ChromiumV2	PBMC	heterogeneous	link	(13)
PBMC-r2 (Drop-seq)	Drop-seq	PBMC	heterogeneous	link	(13)
PBMC-r2 (inDrops)	inDrops	PBMC	heterogeneous	link	(13)
PBMC-r2 (Seq-Well)	Seq-Well	PBMC	heterogeneous	link	(13)
PBMC68k (ChromiumV1)	ChromiumV1	PBMC	heterogeneous	link	
PBMC (ChromiumV3)	ChromiumV3	PBMC	heterogeneous	link	(11)
PBMC (Smart-seq3)	Smart-seq3	PBMC	heterogeneous	link	(14)
Fetal (sci-RNA-seq3)	sci-RNA-seq3	Fetus	heterogeneous	link	(15)
Bone Marrow (CITE-seq)	CITE-seq	Bone Marrow	heterogeneous	link	(4)

Table S1: **List of datasets used in this study.** Raw data can be downloaded from the hyperlinks under 'raw data' column. Similar sample names with '-r1' and '-r2' denote replicates from Ding et al. (13) study.

Sample name	<0.01	>0.01	>0.1	>1	>5	>10	>25	>50	>100
TechCtrl1 (ChromiumV1)	0.000			0.01	0.10	0.70			
TechCtrl2 (ChromiumV1)	0.000	0.000	0.018	0.51		0.93			
TechCtrl (inDrops)			0.000	0.01	0.04	0.13	0.41	0.67	0.93
3T3 (ChromiumV3)	0.002	0.007	0.066	0.49	0.99	1.00	1.00	1.00	
3T3-r1 (CEL-seq2)	0.007	0.007	0.118	0.37	0.84	0.98	1.00	1.00	1.00
3T3-r1 (ChromiumV2)	0.004	0.011	0.196	0.82	1.00	1.00	1.00	1.00	
3T3-r1 (Drop-seq)	0.002	0.001	0.054	0.60	1.00	1.00			
3T3-r1 (inDrops)	0.001	0.001	0.046	0.78	1.00	1.00			
3T3-r1 (sci-RNA-seq)	0.001	0.003	0.056	0.50	0.99	1.00	1.00		
3T3-r2 (CEL-seq2)	0.008	0.009	0.104	0.39	0.77	0.95	1.00	1.00	1.00
3T3-r2 (ChromiumV2)	0.008	0.006	0.143	0.74	1.00	1.00	1.00	1.00	
3T3-r2 (Drop-seq)	0.001		0.039	0.47	0.95	1.00			
3T3-r2 (inDrops)	0.001	0.001	0.029	0.48	0.97	1.00			
3T3-r2 (sci-RNA-seq)	0.005	0.008	0.209	0.87	1.00	1.00	1.00		
HEK (ChromiumV3)	0.003	0.006	0.076	0.51	0.97	1.00	1.00	1.00	1.00
HEK (Smart-seq3)	0.012	0.012	0.172	0.70	0.94	0.99	1.00	1.00	1.00
HEK-r1 (CEL-seq2)		0.008	0.092	0.38	0.76	0.94	1.00	1.00	1.00
HEK-r1 (ChromiumV2)	0.005	0.018	0.158	0.85	1.00	1.00	1.00		1.00
HEK-r1 (Drop-seq)	0.000	0.003	0.038	0.47	1.00	1.00	1.00		
HEK-r1 (inDrops)	0.001	0.001	0.020	0.54	1.00	1.00			
HEK-r1 (sci-RNA-seq)	0.005	0.007	0.076	0.52	0.97	1.00			
HEK-r2 (CEL-seq2)	0.005	0.008	0.085	0.45	0.90	0.98	1.00	1.00	1.00
HEK-r2 (ChromiumV2)	0.006	0.019	0.203	0.91	1.00	1.00	1.00	1.00	
HEK-r2 (Drop-seq)	0.001	0.003	0.059	0.62	1.00	1.00	1.00		
HEK-r2 (inDrops)	0.001	0.003	0.028	0.60	1.00	1.00			
HEK-r2 (sci-RNA-seq)	0.006	0.018	0.265	0.91	1.00	1.00			
HEK-m (CEL-seq2)		0.019	0.136	0.61	0.93	0.99	1.00	1.00	1.00
HEK-m (ChromiumV2)	0.188	0.204	0.675	0.97	1.00	1.00	1.00	1.00	1.00
HEK-m (ChromiumV2_sn)			0.005	0.17	0.73				
HEK-m (ddSeq)	0.222	0.479	0.912	0.99	1.00	1.00			
HEK-m (Drop-seq)	0.006	0.014	0.291	0.98	1.00	1.00			
HEK-m (inDrops)			0.008	0.25	0.69	1.00			
HEK-m (MARS-seq)		0.010	0.209	0.80	0.99	1.00	1.00		
HEK-m (mcSCRB-seq)		0.007	0.334	0.94	1.00	1.00			
HEK-m (Quartz-Seq2)	0.103	0.133	0.525	0.96	1.00	1.00	1.00	1.00	1.00
Cortex-r1 (ChromiumV2)	0.001	0.009	0.124	0.77	1.00	1.00			
Cortex-r1 (DroNc-seq)	0.001	0.007	0.200	0.91	1.00				
Cortex-r1 (sci-RNA-seq)	0.001	0.011	0.258	0.97	1.00	1.00			
Cortex-r2 (ChromiumV2)	0.001	0.006	0.104	0.80	1.00	1.00			
Cortex-r2 (DroNc-seq)	0.002	0.007	0.204	0.92	1.00				
Cortex-r2 (sci-RNA-seq)	0.001	0.010	0.218	0.99	1.00				
Fibroblasts (Smart-seq3)	0.048	0.274	0.799	0.99	1.00	1.00	1.00	1.00	1.00
PBMC-r1 (CEL-seq2)	0.001	0.001	0.017	0.31	0.95	1.00			
PBMC-r1 (ChromiumV2A)	0.000	0.004	0.109	0.87	0.98	1.00			
PBMC-r1 (ChromiumV2B)	0.001	0.002	0.079	0.80	0.98	1.00	1.00		
PBMC-r1 (ChromiumV3)	0.002	0.011	0.145	0.78	1.00	1.00	1.00		
PBMC-r1 (Drop-seq)	0.000	0.002	0.128	0.83	1.00				
PBMC-r1 (inDrops)	0.000	0.003	0.189	0.97					
PBMC-r1 (Seq-Well)	0.001	0.005	0.214	0.96					
PBMC-r2 (CEL-seq2)		0.001	0.013	0.30	0.95	1.00			
PBMC-r2 (ChromiumV2)	0.000	0.001	0.061	0.74	0.98	1.00			
PBMC-r2 (Drop-seq)	0.001	0.004	0.206	0.92	1.00	1.00			
PBMC-r2 (inDrops)	0.001	0.005	0.145	0.96	1.00	1.00			
PBMC-r2 (Seq-Well)	0.000		0.095	0.93					
PBMC68k (ChromiumV1)	0.000	0.003	0.069	0.76	0.98	1.00			
PBMC (ChromiumV3)	0.002	0.011	0.111	0.66	0.95	1.00	1.00	1.00	
PBMC (Smart-seq3)	0.031	0.359	0.894	0.97	1.00	1.00	1.00		
Fetal (sci-RNA-seq3)	0.116	0.617	0.990						
Bone Marrow (CITE-seq)	0.009	0.072	0.562	0.932	0.935	1.00	1.00		

Table S2: **Proportion of non-poisson genes across different gene-mean bins.** Columns indicate non-cumulative gene abundance bins between two consecutive labels (for example, > 1 refers to all genes with mean > 1 and ≤ 5). Each cell entry summarizes the total proportion of genes belonging to a mean abundance bin that were detected to be non-poisson for a dataset.

System	Variable feature rank	$\theta = 100$	$\theta = 10$	sctransform v1	sctransform v2
Bone Marrow	1000	0.204	0.189	0.194	0.290
Bone Marrow	2000	0.305	0.276	0.279	0.427
Bone Marrow	3000	0.367	0.337	0.337	0.536
Cortex	1000	0.263	0.249	0.216	0.292
Cortex	2000	0.415	0.402	0.353	0.474
Cortex	3000	0.544	0.529	0.470	0.586
Fetus	1000	0.210	0.206	0.222	0.287
Fetus	2000	0.356	0.352	0.394	0.455
Fetus	3000	0.453	0.446	0.520	0.542
PBMC	1000	0.190	0.165	0.145	0.282
PBMC	2000	0.292	0.264	0.217	0.459
PBMC	3000	0.385	0.345	0.287	0.571

Table S3: **Proportion of highly variable genes overlapping with marker genes**. Values in columns $\theta = 100$, $\theta = 10$, sctransform v1, and sctransform v2 indicate the median cumulative proportion of genes up to a given rank that overlap with the marker genes in the system (over multiple technologies). Bone Marrow and Fetus, represent datasets with only one technology.

System	Variable feature rank	$\theta = 100$	$\theta = 10$	sctransform v1	sctransform v2
Bone Marrow	1000	611	567	582	871
Bone Marrow	2000	916	829	836	1281
Bone Marrow	3000	1100	1012	1012	1607
Cortex	1000	788	748	650	876
Cortex	2000	1245	1205	1058	1422
Cortex	3000	1633	1586	1408	1758
Fetus	1000	631	618	667	862
Fetus	2000	1068	1056	1182	1366
Fetus	3000	1360	1337	1559	1626
PBMC	1000	569	495	436	847
PBMC	2000	877	793	650	1376
PBMC	3000	1155	1035	860	1712

Table S4: **Number of highly variable genes overlapping with marker genes**. Values in columns $\theta = 100$, $\theta = 10$, sctransform v1, and sctransform v2 indicate the median cumulative number of genes up to a given rank that overlap with the marker genes in the system (over multiple technologies). Bone Marrow and Fetus, represent datasets with only one technology.

References

[1] C. Ahlmann-Eltze and W. Huber, "glmGamPoi: fitting Gamma-Poisson generalized linear models on single cell count data," *Bioinformatics*, vol. 36, pp. 5701–5702, Apr. 2021.

[2] C. Hafemeister and R. Satija, "Analyzing scRNA-seq data with the sctransform and offset models," *https://satijalab.org/pdf/sctransform_offset.pdf*, 2020.

[3] Y. Baran, A. Bercovich, A. Sebe-Pedros, Y. Lubling, A. Giladi, E. Chomsky, Z. Meir, M. Hoichman, A. Lifshitz, and A. Tanay, "MetaCell: analysis of single-cell RNA-seq data using k-nn graph partitions," *Genome Biology*, vol. 20, p. 206, Oct. 2019.

[4] T. Stuart, A. Butler, P. Hoffman, C. Hafemeister, E. Papalexi, W. M. Mauck, 3rd, Y. Hao, M. Stoeckius, P. Smibert, and R. Satija, "Comprehensive integration of Single-Cell data," *Cell*, vol. 177, pp. 1888–1902.e21, June 2019.

[5] M. I. Love, W. Huber, and S. Anders, "Moderated estimation of fold change and dispersion for RNA-seq data with DESeq2," *Genome Biology*, vol. 15, no. 12, p. 550, 2014.

[6] G. Finak, A. McDavid, M. Yajima, J. Deng, V. Gersuk, A. K. Shalek, C. K. Slichter, H. W. Miller, M. J. McElrath, M. Prlic, P. S. Linsley, and R. Gottardo, "MAST: a flexible statistical framework for assessing transcriptional changes and characterizing heterogeneity in single-cell RNA sequencing data," *Genome Biology*, vol. 16, p. 278, Dec. 2015.

[7] F. Wilcoxon, "Individual comparisons by ranking methods," in *Breakthroughs in Statistics: Methodology and Distribution* (S. Kotz and N. L. Johnson, eds.), pp. 196–202, New York, NY: Springer New York, 1992.

[8] H. L. Crowell, C. Soneson, P.-L. Germain, D. Calini, L. Collin, C. Raposo, D. Malhotra, and M. D. Robinson, "Muscat detects subpopulation-specific state transitions from multi-sample multi-condition single-cell transcriptomics data," *Nature Communications*, vol. 11, no. 1, pp. 1–12, 2020.

[9] E. Mereu, A. Lafzi, C. Moutinho, C. Ziegenhain, D. J. McCarthy, A. Álvarez-Varela, E. Batlle, Sagar, D. Grün, J. K. Lau, S. C. Boutet, C. Sanada, A. Ooi, R. C. Jones, K. Kaihara, C. Brampton, Y. Talaga, Y. Sasagawa, K. Tanaka, T. Hayashi, C. Braeuning, C. Fischer, S. Sauer, T. Trefzer, C. Conrad, X. Adiconis, L. T. Nguyen, A. Regev, J. Z. Levin, S. Parekh, A. Janjic, L. E. Wange, J. W. Bagnoli, W. Enard, M. Gut, R. Sandberg, I. Nikaido, I. Gut, O. Stegle, and H. Heyn, "Benchmarking single-cell RNA-sequencing protocols for cell atlas projects," *Nature Biotechnology*, vol. 38, pp. 747–755, June 2020.

[10] V. Svensson, K. N. Natarajan, L. H. Ly, R. J. Miragaia, and others, "Power analysis of single-cell RNA-sequencing experiments," *Nature*, 2017.

[11] V. Svensson, "Droplet scRNA-seq is not zero-inflated," *Nature Biotechnology*, vol. 38, pp. 147–150, Feb. 2020.

[12] A. M. Klein, L. Mazutis, I. Akartuna, N. Tallapragada, A. Veres, V. Li, L. Peshkin, D. A. Weitz, and M. W. Kirschner, "Droplet barcoding for single-cell transcriptomics applied to embryonic stem cells," *Cell*, vol. 161, pp. 1187–1201, May 2015.

[13] J. Ding, X. Adiconis, S. K. Simmons, M. S. Kowalczyk, C. C. Hession, N. D. Marjanovic, T. K. Hughes, M. H. Wadsworth, T. Burks, L. T. Nguyen, J. Y. H. Kwon, B. Barak, W. Ge, A. J. Kedaigle, S. Carroll, S. Li, N. Hacohen, O. Rozenblatt-Rosen, A. K. Shalek, A.-C. Villani, A. Regev, and J. Z. Levin, "Systematic comparison of single-cell and single-nucleus RNA-sequencing methods," *Nature Biotechnology*, vol. 38, pp. 737–746, June 2020.

[14] M. Hagemann-Jensen, C. Ziegenhain, P. Chen, D. Ramsköld, G.-J. Hendriks, A. J. M. Larsson, O. R. Faridani, and R. Sandberg, "Single-cell RNA counting at allele and isoform resolution using smart-seq3," *Nature Biotechnology*, vol. 38, pp. 708–714, June 2020.

[15] J. Cao, D. R. O'Day, H. A. Pliner, P. D. Kingsley, M. Deng, R. M. Daza, M. A. Zager, K. A. Aldinger, R. Blecher-Gonen, F. Zhang, M. Spielmann, J. Palis, D. Doherty, F. J. Steemers, I. A. Glass, C. Trapnell, and J. Shendure, "A human cell atlas of fetal gene expression," *Science*, vol. 370, Nov. 2020.
