# Supplementary material for: Comparison and evaluation of statistical error models for scRNA-seq
Source: Genome Biol. 2022 Jan 18;23:27. doi: 10.1186/s13059-021-02584-9 (PMC8764781; doi:10.1186/s13059-021-02584-9)
Supplement: Supplementary file 2 — Additional file 2 Review history. [file 13059_2021_2584_MOESM2_ESM.docx]

Review History

**First round of review**

**Reviewer 1**

**Are you able to assess all statistics in the manuscript, including the appropriateness of statistical tests used?**

Yes, and I have assessed the statistics in my report.

**Comments to author:**

In this work, Choudhary and Satija performed comprehensive evaluation of statistical models for single cell RNA-seq data. The modeling of scRNA-seq count data is an important first step in scRNA-seq data analysis and lays the foundation for many downstream analyses include cell clustering, pseudotime construction, differential expression, etc. The authors fit different models on 58 datasets from different technologies and made some general conclusion and recommendation.

Overall, the paper is well written, and the results are well presented. However, a major problem for this work is the lack of novelty. Below I provide some specific comments:

- There are 3 main conclusions in this work: (1) Poisson model is inappropriate; (2) Negative binomial with gene-specific dispersion is preferred; and (3) Dispersion varies as a function as mean expression level. All these are well known facts since the bulk RNA-seq days, and well recognized in scRNA-seq community. The analyses in this work, albeit nicely conducted, just confirmed these findings, which have been reported repeatedly before.

- The authors also developed a procedure for estimating parameters and showed improved goodness of fit for the data. However, in order to demonstrate that the procedure is useful, authors ought to show that the new procedure can help downstream analysis such as cell clustering. Otherwise, the improved estimation won't have any impact on the data analysis.

- I don't quite agree with the authors' decomposition of cell-to-cell variation in the conclusion section. I don't think differences among cell types and states are considered "variation". They are "fixed effects" in statistical jargon. Their third component is "variation", or "random effect". From this perspective, I think all analyses in this work should be performed conditioned on the cell types, i.e., first cluster the cells, then analyze their dispersion within the same cell type. Of course, I don't expect any difference in the major conclusion. Moreover, I think the sentence "Our analyses suggest a third level of variation that should be accounted for …" is slightly overstated, since it's known that the over-dispersion exists even within the same cell type.

- One minor comment: citation 10 is an "author correction". It might be better to cite the original paper?

**Reviewer 2**

**Are you able to assess all statistics in the manuscript, including the appropriateness of statistical tests used?**

Yes, and I have assessed the statistics in my report.

**Comments to author:**

Normalization and variance stabilization of single cell RNA sequencing data are crucial to downstream analysis such as dimension reduction and differential expression analysis. In this manuscript, the authors analyzed the distributions of genes in 58 single cell RNA sequencing datasets comprehensively and evaluated the performance of different error models for normalization and variance stabilization. Furthermore, they modified the model and the regularization procedure of sctransform and proposed sctransform v2 which improves the robustness of NB parameter estimates in sctransform v1 and has more computational efficiency. The paper is well written and since sctransform v1 has already be widely used, I believe the new version of sctransform will be very helpful for single cell RNA sequencing data analysis. But I still have the following concern:

The authors modified the sctransform v1 according to three conclusions obtained from the evaluation of 58 datasets. a. Most of genes with higher expression levels failed the Poisson goodness-of-fit test and should be modeled by a negative binomial model. b the level of overdispersion varies across datasets. c. the overdispersion varies as a function of gene expression levels. For a, recent study (ref 25) suggests method development should start with a Poisson model, but the results showed in this manuscript especially in heterogeneous datasets are quite convincing. For b and c, however, the authors need show more evidences to prove the NB model with different inverse overdispersion parameters can actually improve the performance compared to the fixed parameter model (as ref 22 proposed). And it is also necessary to evaluate the effect of the sctransform v2 on downstream processing such as variable genes selection, or differential expression analysis. So, I suggest the authors evaluate the effects of different models (sequencing depth normalization followed by log-transformation, sctransform v2, inverse overdispersion parameter fixed NB and maybe also Poisson model) on downstream analysis. That will be more convincing than just statistical analysis showed in Fig 2.

Minor: The caption of Fig 1E: "Same as (A)" should be "Same as (B)"

**Authors Response**

**Point-by-point responses to the reviewers’ comments:**

*We thank both reviewers for their clear and constructive comments on our submission. In our re-vised manuscript, we have included new analyses to address each suggestion. We believe these revisions have substantially improved the manuscript, and in particular, will help readers to appre¬ciate the comparative advantages of our proposed modeling and normalization approach. While we address each comment in a point-by-point response, we also summarize our key revisions below.*

*Additional benchmarking for sctransform v2*

*Each of the reviewers expressed enthusiasm for our work, but requested additional benchmark-ing analyses to demonstrate that our proposed framework not only best represents the statistical properties of scRNA-seq data as we showed in our original submission, but also improves per-formance in downstream analyses. Reviewers asked for benchmarking sctransform v2 against our previous version (sctransform v1), standard log-normalization, and models with fixed negative binomial parameters. We agree with these comments and have added two key analyses to our revised manuscript:*

*1)	Benchmarking of variable gene identification: A key downstream step of single-cell analyt¬ical workflows involves the identification of genes that exhibit high variation across single-cells, which are identified prior to clustering, and are used as input to dimensional reduction analyses. To perform benchmarking analyses, we used a strategy motivated by (1), which considers the overlap between an identified set of variable features, and a set of features that are differentially expressed across cell types.*

*We included this benchmarking analysis in our revised manuscript. As shown in our revised Figure 4 and Supplementary Figure 22 (and reproduced below), we show that sctransform v2 substan¬tially improves the identification of marker genes as variable features, across a range of biological systems, sequencing depths, and technologies. We also show that sctransform v2 identifies vari¬able features that span a range of expression levels, while sctransform v1 erroneously tends to identify genes with extremely low abundance, while models with fixed θ values tend to be biased towards genes with extremely high abundance.*

*2)	Benchmarking differential expression after normalization: A second key downstream ana¬lytical step represents the identification of differentially expressed (DE) genes. Previous work (2) has shown that the performance of this task is heavily dependent on the normalization procedure. The same manuscript found that when performing DE across datasets that had been normal¬ized independently, methods based on Pearson residuals tended to identify a high proportion of false-positives. In sctransform v2, we therefore devised an alternative procedure to perform DE on ‘corrected’ UMI counts that can be extracted from the regularized negative binomial model. We benchmarked our revised procedure to identify DE genes either across datasets (for example, representing multiple conditions), or to identify DE genes between different subpopulations within an individual dataset.*

*We followed the benchmarking workflow in (2), and used the muscat tool to simulate scRNA-seq datasets from multiple conditions with variable proportions of DE genes. We then compared the results of differential expression, comparing the results with ground-truth from the simulations. We benchmarked sctransform v2 against our previous work (sctransform v1), and two size-factor based normalization methods (scran, and log-normalization). The results are included in our revised Figure 4 and reproduced below. We show that sctransform v2 consistently identifies DE genes at a higher true positive rate (TPR) for the same false-discovery rate (FDR) threshold. The improvement is especially pronounced when there are substantial differences in sequencing depth across conditions. In these cases, we find that existing workflows (including sctransform v1) intro¬duce multiple false-positive calls during DE, but these are removed using sctransform v2.*

*Taken together, we conclude that sctransform v2 not only includes a modeling approach that best represents the statistical properties of scRNA-seq data, but it also improves performance in key downstream analytical tasks across a wide range of datasets.*

Reviewer #1

Comment #1: There are 3 main conclusions in this work: (1) Poisson model is inappropriate; (2) Negative binomial with gene-specific dispersion is preferred; and (3) Dispersion varies as a function as mean expression level. All these are well known facts since the bulk RNA-seq days, and well recognized in scRNA-seq community. The analyses in this work, albeit nicely conducted, just confirmed these findings, which have been reported repeatedly before.

*Response: We agree with the reviewer that our findings are consistent with previous studies of bulk RNA-seq data, as we cite throughout the manuscript. However, we respectfully disagree that these conclusions are well-accepted in the single-cell field. For example, while a wide variety of statistical models have been proposed and implemented for scRNA-seq (3–7), only sctransform utilizes a negative binomial model with a gene-specific overdispersion that is regularized as a function of average expression level. In particular, two recent papers [(6), (7)], propose statistical modeling frameworks for scRNA-seq that conflict with the recommendations in this manuscript.*

*A key contribution of our manuscript is to analyze datasets across a broad range of sequencing depths and technologies. Specifically, we find that fixed parameter estimates may appear to be appropriate for shallow datasets, but that more flexible parameter estimation procedures are re-quired as datasets increase in sequencing depth. These findings yield clear recommendations to the scRNA-seq community, which are implemented in sctransform v2.*

*In our revised manuscript, we not only demonstrate that sctransform v2 best represents the statis¬tical properties of scRNA-seq datasets across a wide breadth of datasets, but also that it improves on key downstream analytical steps including highly variable gene identification and differential expression. Taken together, we believe that our work makes a timely, novel, and important contri¬bution to the single-cell field.*

Comment #2: The authors also developed a procedure for estimating parameters and showed improved goodness of fit for the data. However, in order to demonstrate that the procedure is useful, authors ought to show that the new procedure can help downstream analysis such as cell clustering. Otherwise, the improved estimation won’t have any impact on the data analysis.

*Response: We thank the reviewer for their suggestion, and agree that it is important to benchmark sctransform v2 on downstream analyses that are dependent on normalization and preprocessing. As described previously in our response, we benchmarked our method on two key tasks: variable gene selection, and differential expression. We chose these tasks because they are essential to nearly all single-cell analyses. In addition, we felt that robust and benchmarking procedures have been described for this task. For example, for variable gene selection, we implemented the benchmarking procedure motivated by (1). For differential expression, we implemented the benchmarking procedure introduced by (2).*

*For both variable gene selection and differential expression, we found that sctransform v2 out-performs existing methods (Response Figures 1 and 2). This includes a substantial improvement over our previous work (sctransform v1). The full results are described in our revised manuscript (Figure 4 and Supplementary Figures 17 and 22), and summarized earlier in this response. We thank the reviewer again for this suggestion and believe that these analyses will be valuable for readers of our manuscript, and users of our software package.*

*We chose not to proceed further and perform extensive benchmarking on cell clustering, as we felt that there were not robust benchmarking procedures for this task. This reflects a fundamental limitation and difficulty in obtaining ‘ground-truth’ labels for scRNA-seq clustering that can be used for benchmarking. This is particularly difficult as we wished to benchmark on tasks that spanned a range of technologies and systems. We therefore chose to focus our benchmarking analyses on variable gene selection and differential expression.*

*Response Figure 1: Taken from Figure 4. A) Proportion overlap (median) of variable features with marker genes across heterogeneous datasets using fixed θ = {100, 10}, our original regulariza¬tion procedure from (5) (v1 regularization), and using modified regularization (v2 regularization). Marker genes were identified using unsupervised clustering on log normalized data using presto (8).*

*In addition, using two complementary analyses, we show the usefulness of our approach at dif-ferential expression (DE) analysis. On simulated datasets with varying proportion of DE genes, our approach results in the highest true positive rate. In a complementary analysis, by performing differential expression on two biologically similar population with different sequencing depths, we show that our approach has highest specificity (Response Figure 2).*

Comment #3: I don’t quite agree with the authors’ decomposition of cell-to-cell variation in the conclusion section. I don’t think differences among cell types and states are considered "variation".

*Response Figure 2: Taken from Figure 4. C) Differential expression benchmarking. Observed overall true positive rate (TPR) and false discovery rate (FDR) values at FDR cutoffs of 1%, 5%, and 10% using a Wilcoxon test. Titles indicate simulated proportion of DE genes. D) Number of differential expression genes identified between two groups of biological identical NK cells (PBMC Smart-seq3) where one group was randomly downsampled to 20% sequencing depth.*

They are "fixed effects" in statistical jargon. Their third component is "variation", or "random effect". From this perspective, I think all analyses in this work should be performed conditioned on the cell types, i.e., first cluster the cells, then analyze their dispersion within the same cell type. Of course, I don’t expect any difference in the major conclusion. Moreover, I think the sentence "Our analyses suggest a third level of variation that should be accounted for . . . " is slightly overstated, since it’s known that the over-dispersion exists even within the same cell type.

*Response: We thank the reviewer for this comment, and have extensively revised our language in the discussion sections. In particular, we agree with the observation that difference among cell type and state are "fixed effects". We have reworded the section to reflect that our findings are consistent with previous reports of intrinsic variation, and emphasized our broader point, which is that such noise is only detectable at more pronounced sequencing depths. We also agree that it is important to study ‘random effects’ within a ‘homogeneous’ cell type, which is why examine multiple scRNA-seq datasets of cell lines (as opposed to heterogeneous tissues) throughout the manuscript. We felt this was a more accessible way to study intrinsic variation instead of repeating our analyses on each cluster of cells (across 59 datasets, repeating each analysis for an individual cluster would result in thousands of new analyses).*

Comment #4: One minor comment: citation 10 is an "author correction". It might be better to cite the original paper?

*Response: Thanks for bringing this to our attention. We have corrected the citation.*

Reviewer #2

Comment #5: The authors modified the sctransform v1 according to three conclusions obtained from the evaluation of 58 datasets. a. Most of genes with higher expression levels failed the Poisson goodness-of-fit test and should be modeled by a negative binomial model. b the level of overdispersion varies across datasets. c. the overdispersion varies as a function of gene ex-pression levels. For a, recent study (ref 25) suggests method development should start with a Poisson model, but the results showed in this manuscript especially in heterogeneous datasets are quite convincing. For b and c, however, the authors need show more evidences to prove the NB model with different inverse overdispersion parameters can actually improve the performance compared to the fixed parameter model (as ref 22 proposed). And it is also necessary to evaluate the effect of the sctransform v2 on downstream processing such as variable genes selection, or differential expression analysis. So, I suggest the authors evaluate the effects of different models (sequencing depth normalization followed by log-transformation, sctransform v2, inverse overdis-persion parameter fixed NB and maybe also Poisson model) on downstream analysis. That will be more convincing than just statistical analysis showed in Fig 2.

*Response: We thank the reviewer for their suggestion, and agree that it is important to benchmark sctransform v2 on downstream analyses that are dependent on normalization and preprocessing. As described previously in our response, we benchmarked our method on two key tasks: variable gene selection, and differential expression. We chose these tasks because they are essential to nearly all single-cell analyses. In addition, we felt that robust and benchmarking procedures have been described for this task. For example, for variable gene selection, we implemented the benchmarking procedure motivated by (1). For differential expression, we implemented the benchmarking procedure introduced by (2).*

*For both variable gene selection and differential expression, we found that sctransform v2 out-performs existing methods (Response Figures 1 and 2). This includes a substantial improvement over our previous work (sctransform v1). The full results are described in our revised manuscript (Figure 4 and Supplementary Figures 17 and 22), and summarized earlier in this response. We thank the reviewer again for this suggestion and believe that these analyses will be valuable for readers of our manuscript, and users of our software package.*

Comment #6: Minor: The caption of Fig 1E: "Same as (A)" should be "Same as (B)"

*Response: We thank the reviewer for pointing this out. We have corrected the legend.*

*References*

*[1]	T. S. Andrews and M. Hemberg, “M3Drop: dropout-based feature selection for scRNASeq,” Bioinformatics, vol. 35, pp. 2865–2867, Aug. 2019.*

*[2]	H. L. Crowell, C. Soneson, P.-L. Germain, D. Calini, L. Collin, C. Raposo, D. Malhotra, and M. D. Robinson, “muscat detects subpopulation-specific state transitions from multi-sample multi-condition single-cell transcriptomics data,” Nature Communications, vol. 11, p. 6077, Nov. 2020.*

*[3]	D. Grün, L. Kester, and A. van Oudenaarden, “Validation of noise models for single-cell tran-scriptomics,” Nature Methods, vol. 11, pp. 637–640, June 2014.*

*[4]	D. Risso, F. Perraudeau, S. Gribkova, S. Dudoit, and J. P. Vert, “ZINB-WaVE: A general and flexible method for signal extraction from single-cell RNA-seq data,” bioRxiv, 2017.*

*[5]	C. Hafemeister and R. Satija, “Normalization and variance stabilization of single-cell RNA-seq data using regularized negative binomial regression,” Genome Biology, vol. 20, p. 296, Dec. 2019.*

*[6]	J. Lause, P. Berens, and D. Kobak, “Analytic pearson residuals for normalization of single-cell RNA-seq UMI data,” Genome Biology, vol. 22, p. 258, Sept. 2021.*

*[7]	A. Sarkar and M. Stephens, “Separating measurement and expression models clarifies con-fusion in single-cell RNA sequencing analysis,” Nature Genetics, vol. 53, pp. 770–777, May 2021.*

*[8]	I. Korsunsky, A. Nathan, N. Millard, and S. Raychaudhuri, “Presto scales Wilcoxon and auROC analyses to millions of observations,” bioRxiv, p. 653253, 2019.*

**Second round of review**

**Reviewer 1**

The revised manuscript is greatly improved. However, I still have following questions and comments:

- To save the reviewer some efforts, please highlight the modified parts using a different color in the manuscript. The authors mentioned that they have substantially modified the language. Without highlighting, it's impossible for the reviewer to see the modification other than to compare with the original version.

- For comparison of DE analysis: it's not clear why the authors chose Wilcoxan test, since there are so many DE tools readily available for scRNA-seq. For example, MAST seems to be a good choice since it also takes log TPM as input. Please also compare with DESeq2 using raw counts as input. Even though it's not designed for scRNA-seq, it uses similar data modeling technique (negative binomial, gene-specific overdispersion that is related to average expression). The authors really need to show methodological novelty and demonstrate that this model provides advantage over existing ones.

- The DE analyses are completely based on simulation. It's important to test on real data. Of course, the gold standard in real data is hard to obtain. But there are data generated for the scnorm paper (PMID: 28418000), where H1 and H9 cells are profiled using very different sequencing depth. These data are great resources for benchmarking methods. Authors can validate their results on those datasets, for example, to test on H1 and H9 data with different depths. This is essentially the same as the simulated "null" test (Fig 4D), but much more convincing.

**Reviewer 2**

The authors evaluated sctranform v2 on variable genes selection and differential expression analysis and showed that the performance of sctranform v2 is improved in downstream analysis in reivsed manuscript. The Authors have addressed all of my concerns with the original manuscript.

**Authors Response**

**Point-by-point responses to the reviewers’ comments:**

Reviewer #1

Comments: Revised manuscript is greatly improved. However, I still have the following questions and comments:

·	To save the reviewer some efforts, please highlight the modified parts using a different color in the manuscript. The authors mentioned that they have substantially modified the language. Without highlighting, it’s impossible for the reviewer to see the modification other than to compare with the original version.

·	For comparison of DE analysis: it’s not clear why the authors chose Wilcoxon test, since there are so many DE tools readily available for scRNA-seq. For example, MAST seems to be a good choice since it also takes log TPM as input. Please also compare with DESeq2 using raw counts as input. Even though it’s not designed for scRNA-seq, it uses similar data modeling technique (negative binomial, gene-specific overdispersion that is related to aver¬age expression). The authors really need to show methodological novelty and demonstrate that this model provides advantage over existing ones.

·	The DE analyses are completely based on simulation. It’s important to test on real data. Of course, the gold standard in real data is hard to obtain. But there are data generated for the scnorm paper (PMID: 28418000), where H1 and H9 cells are profiled using very different sequencing depth. These data are great resources for benchmarking methods. Authors can validate their results on those datasets, for example, to test on H1 and H9 data with different depths. This is essentially the same as the simulated "null" test (Fig 4D), but much more convincing.

*Response:*

*We appreciate the reviewer’s positive comments regarding our last round of revision.*

*We agree that it is important to show that our improvements for single-cell differential expression (DE) are not unique to a single test. We highlight that sctransform v2 does not propose a new sta¬tistical test for DE, but instead proposes an improved normalization strategy, returning corrected counts that can be used as input for different statistical tests. We had originally performed bench-marking using the Wilcoxon test as this makes no parametric assumptions, and has been shown to exhibit good performance (1).*

*As requested by the reviewer, we repeated our benchmarking analyses using the MAST (2) DE test. We observed identical results when using either MAST or Wilcoxon, and have added Sup-plementary Figure 23 to show this (reproduced below).*

*The reviewer also suggests that we perform a ‘null’ benchmarking experiment (where none or very few DE genes are expected) based on real data in addition to simulation. We utilized data from (3), which the same biological system (HEK293 cells), but profiled with two different technologies (Drop-Seq and Quartz-Seq2) that exhibit starkly different sequencing depths. We did not use the specific datasets suggested by the reviewer (PMID: 28418000 (4)), as these datasets were generated with the Fluidigm C1 system and did not include unique molecular identifiers (UMIs).*

*We have included our results in a new Supplementary Figure 25 (reproduced below). These results are fully concordant with our simulation data, and show that normalization with sctransform v2 returns substantially fewer false positives in this negative control. This result is consistent regardless of which method for DE testing is utilized.*

*The reviewer also asked us to benchmark against DESeq2 (5) as well. We note that DESeq2 was not originally designed for the analysis of scRNA-seq, or other sparse RNA profiling technologies. As discussed by Van den Berge et al. (6) and Lun et al. (7), the extensive zero values in scRNA-seq pose challenges for size factor estimation in DESeq2. We therefore computed size factors using scran (7), and then proceed with the DESeq2 workflow. When using these modifications, we found that DESeq2 and sctransform v2 were the best-performing methods when analyzing sim¬ulated datasets, particularly when there were no major differences in sequencing depth between experiments. However, when running benchmarks using datasets from the literature as suggested by the reviewer, we found that DESeq2 run with unnormalized raw counts identified a large num¬ber of false positives (12, 270), similar to standard normalization methods, but substantially higher than when using sctransform v2 corrected counts as input (870 genes).*

*We conclude that sctransform v2 returns corrected counts that can robustly applied to multiple differential expression tests, and that minimize false positive results even when there are significant differences in sequencing depth across experiments and conditions.*

*These revisions are included in the new verison of the manuscript. As requested by the reviewer, we have ensured that all textual changes in the manuscript are highlighted. Please note that we also highlight changes that were included in the first round of revision (compared to the original submission).*

*References*

*[1]	C. Soneson and M. D. Robinson, “Bias, robustness and scalability in single-cell differential expression analysis,” Nat. Methods, vol. 15, pp. 255–261, Apr. 2018.*

*[2]	G. Finak, A. McDavid, M. Yajima, J. Deng, V. Gersuk, A. K. Shalek, C. K. Slichter, H. W. Miller, M. J. McElrath, M. Prlic, P. S. Linsley, and R. Gottardo, “MAST: a flexible statistical framework for assessing transcriptional changes and characterizing heterogeneity in single-cell RNA sequencing data,” Genome Biology, vol. 16, p. 278, Dec. 2015.*

*[3]	E. Mereu, A. Lafzi, C. Moutinho, C. Ziegenhain, D. J. McCarthy, A. Álvarez-Varela, E. Batlle, Sagar, D. Grün, J. K. Lau, S. C. Boutet, C. Sanada, A. Ooi, R. C. Jones, K. Kaihara, C. Bramp¬ton, Y. Talaga, Y. Sasagawa, K. Tanaka, T. Hayashi, C. Braeuning, C. Fischer, S. Sauer, T. Tre-fzer, C. Conrad, X. Adiconis, L. T. Nguyen, A. Regev, J. Z. Levin, S. Parekh, A. Janjic, L. E. Wange, J. W. Bagnoli, W. Enard, M. Gut, R. Sandberg, I. Nikaido, I. Gut, O. Stegle, and H. Heyn, “Benchmarking single-cell RNA-sequencing protocols for cell atlas projects,” Nature Biotechnology, vol. 38, pp. 747–755, June 2020.*

*[4]	R. Bacher, L.-F. Chu, N. Leng, A. P. Gasch, J. A. Thomson, R. M. Stewart, M. Newton, and*

*C. Kendziorski, “SCnorm: robust normalization of single-cell RNA-seq data,” Nat. Methods, vol. 14, pp. 584–586, June 2017.*

*[5]	M. I. Love, W. Huber, and S. Anders, “Moderated estimation of fold change and dispersion for RNA-seq data with DESeq2,” Genome Biology, vol. 15, no. 12, p. 550, 2014.*

*[6]	K. Van den Berge, F. Perraudeau, C. Soneson, M. I. Love, D. Risso, J.-P. Vert, M. D. Robinson, S. Dudoit, and L. Clement, “Observation weights unlock bulk RNA-seq tools for zero inflation and single-cell applications,” Genome Biol., vol. 19, p. 24, Feb. 2018.*

*[7]	A. T. L. Lun, K. Bach, and J. C. Marioni, “Pooling across cells to normalize single-cell RNA sequencing data with many zero counts,” Genome Biology, vol. 17, p. 75, Apr. 2016.*

*[8]	H. L. Crowell, C. Soneson, P.-L. Germain, D. Calini, L. Collin, C. Raposo, D. Malhotra, and M. D. Robinson, “muscat detects subpopulation-specific state transitions from multi-sample multi-condition single-cell transcriptomics data,” Nature Communications, vol. 11, p. 6077, Nov. 2020.*

**Third round of review**

**Reviewer 1**

All my concerns have been satisfactorily addressed. Recommend for acceptance.
